# Modulating Visuomotor Sequence Learning by Repetitive Transcranial Magnetic Stimulation: What Do We Know So Far?

**DOI:** 10.3390/jintelligence11100201

**Published:** 2023-10-13

**Authors:** Laura Szücs-Bencze, Teodóra Vékony, Orsolya Pesthy, Nikoletta Szabó, Tamás Zsigmond Kincses, Zsolt Turi, Dezso Nemeth

**Affiliations:** 1 Department of Neurology, University of Szeged, Semmelweis utca 6, H-6725 Szeged, Hungary; 2 Centre de Recherche en Neurosciences de Lyon CRNL U1028 UMR5292, INSERM, CNRS, Université Claude Bernard Lyon 1, 95 Boulevard Pinel, F-69500 Bron, France; 3 Doctoral School of Psychology, ELTE Eötvös Loránd University, Izabella utca 46, H-1064 Budapest, Hungary; 4 Brain, Memory and Language Research Group, Institute of Cognitive Neuroscience and Psychology, Research Centre for Natural Sciences, Magyar Tudósok Körútja 2, H-1117 Budapest, Hungary; 5 Institute of Psychology, ELTE Eötvös Loránd Universiry, Izabella utca 46, H-1064 Budapest, Hungary; 6 Department of Radiology, University of Szeged, Semmelweis utca 6, H-6725 Szeged, Hungary; 7 Department of Neuroanatomy, Institute of Anatomy and Cell Biology, Faculty of Medicine, University of Freiburg, Albertstrasse 17, D-79104 Freiburg, Germany; 8 BML-NAP Research Group, Institute of Psychology & Institute of Cognitive Neuroscience and Psychology, ELTE Eötvös Loránd University & Research Centre for Natural Sciences, Damjanich utca 41, H-1072 Budapest, Hungary

**Keywords:** non-invasive brain stimulation, sequence learning, repetitive TMS, DLPFC

## Abstract

Predictive processes and numerous cognitive, motor, and social skills depend heavily on sequence learning. The visuomotor Serial Reaction Time Task (SRTT) can measure this fundamental cognitive process. To comprehend the neural underpinnings of the SRTT, non-invasive brain stimulation stands out as one of the most effective methodologies. Nevertheless, a systematic list of considerations for the design of such interventional studies is currently lacking. To address this gap, this review aimed to investigate whether repetitive transcranial magnetic stimulation (rTMS) is a viable method of modulating visuomotor sequence learning and to identify the factors that mediate its efficacy. We systematically analyzed the eligible records (n = 17) that attempted to modulate the performance of the SRTT with rTMS. The purpose of the analysis was to determine how the following factors affected SRTT performance: (1) stimulated brain regions, (2) rTMS protocols, (3) stimulated hemisphere, (4) timing of the stimulation, (5) SRTT sequence properties, and (6) other methodological features. The primary motor cortex (M1) and the dorsolateral prefrontal cortex (DLPFC) were found to be the most promising stimulation targets. Low-frequency protocols over M1 usually weaken performance, but the results are less consistent for the DLPFC. This review provides a comprehensive discussion about the behavioral effects of six factors that are crucial in designing future studies to modulate sequence learning with rTMS. Future studies may preferentially and synergistically combine functional neuroimaging with rTMS to adequately link the rTMS-induced network effects with behavioral findings, which are crucial to develop a unified cognitive model of visuomotor sequence learning.

## 1. Introduction

Sequence learning is a fundamental ability of the human brain. It forms the basis of many cognitive, social, and motor skills (Bergstrom et al. 2012; Lieberman 2000; Nemeth et al. 2011). Acquiring the rules of a language, learning to play an instrument, or recognizing the habits of a loved one—all occur by becoming sensitive to sequences of events. The Serial Reaction Time Task (SRTT) is one of the most popular paradigms in visuomotor sequence learning research; it has been used in ~2500 different experimental psychology, clinical, and cognitive neuroscience studies. However, if someone wishes to design a non-invasive brain stimulation study involving the SRTT, they may not be aware of the factors to consider. There is no available list of these factors, which makes planning an experiment challenging. In the current study, our aim was to explore the effect of repetitive transcranial magnetic stimulation (rTMS) on SRTT performance and to describe six key factors to consider when combining rTMS with the SRTT. Thus, this concise review is primarily intended for researchers involved in TMS and sequence learning studies. Nevertheless, it may also be of interest to individuals working in the realms of motor learning, perceptual learning, or, more broadly, memory and decision making, as they can glean valuable insights from the research presented on the neurocognition of sequential processes that play a pivotal role in human cognition.

Previous studies have used observational and interventional methods to better understand the functional role of the brain regions and networks that underlie sequence learning. On the one hand, neuroimaging studies have revealed that particular brain areas are engaged during the learning and retrieval of sequence information. These include the primary motor cortex (M1), supplementary motor area (SMA), dorsolateral prefrontal cortex (DLPFC), parietal cortex, basal ganglia, and cerebellum (Dahms et al. 2020; Daselaar et al. 2003; Dharani 2015; Foerde and Poldrack 2009; Keele et al. 2003; Khatibi et al. 2022; Poldrack et al. 2005; Seidler et al. 2005; Willingham et al. 2002). Whereas neuroimaging methods allow for the characterization of the spatial and temporal features of neuronal activity, interventional methods can modulate brain activity (e.g., cortical excitability), which can facilitate the drawing of causal inferences about the functional role of brain regions and networks (Bergmann and Hartwigsen 2020). Repetitive transcranial magnetic stimulation (rTMS) is an increasingly used non-invasive brain stimulation (NIBS) tool to examine the functional role of cortical areas and brain networks (Bergmann and Hartwigsen 2020; Pascual-Leone et al. 1991). In addition to neuroimaging methods, rTMS might contribute to a better understanding of the functional and neural underpinnings of visuomotor sequence learning. However, can rTMS effectively alter sequence learning performance in humans? If yes, which stimulation parameters govern its efficacy? If we could answer these questions, we would gain a better mechanistic understanding of the functional involvement of brain regions in visuomotor sequence learning. Furthermore, this improved understanding could lead to optimized rTMS protocols and study designs that may effectively enhance learning performance in healthy participants and patients. To answer these questions, we reviewed those studies that have examined the cognitive effects of rTMS on visuomotor sequence learning using the SRTT.

This review summarizes the existing results and outlines those factors that should be considered when designing a new rTMS-SRTT study. We focus on the SRTT and its various versions, as this paradigm offers a broader understanding of sequence learning by enabling the calculation of measures across different levels of learning (e.g., acquire simple deterministic sequences vs. higher-order associations; see later in section *Different Variations of the SRTT*). Additionally, the choice to focus on the SRTT paradigm is driven by the wide range of neural backgrounds seen in other paradigms, which results in significant variance in the effects of rTMS. On the one hand, the goal of the present review is not to provide an ultimate practice guideline listing the best stimulation and task parameters. Instead, it aims to provide a comprehensive overview of six important factors that one needs to consider when combining rTMS with the SRTT. To do so, we will highlight their roles in visuomotor sequence learning and their hidden variability in the literature. Therefore, the present review can facilitate the design of subsequent experiments by pointing out crucial factors and parameters guided based on prior evidence-based findings. Finally, exciting and useful avenues for future research are discussed.

### 1.1. Different Variations of the SRTT

The SRTT has aimed to measure unconscious, implicit learning processes. In the classical SRTT (Nissen and Bullemer 1987), the participant must respond to a visual stimulus that appears in one of four horizontal locations by pressing the corresponding button on a response device as quickly and accurately as possible. The stimuli follow each other in a predetermined order, forming a repeating sequence (e.g., ‘2-3-1-4-3-2-4-1-3-4-2-1’, where numbers represent the four possible locations). In the control task, the elements appear in random positions. Compared to the random sequences, participants respond gradually faster and with higher accuracy in the repeating sequences throughout learning, which shows the acquisition of the sequence order.

This classic version of the SRTT measures the implicit learning of a deterministic sequence (Robertson 2007). In the last few decades, several new versions of this task have emerged, which can differ in three fundamental dimensions: (1) whether the participants are aware of the sequence structure or not (explicit or implicit task), (2) whether an element of the sequence predicts the next element with 100% or with a certain probability (deterministic or probabilistic sequence), and (3) whether the preceding or the n-2 element predicts the occurrence of a given element of the sequence (first-order conditional or second-order conditional sequence). As these diverse sequence types assume different cognitive processes (Prashad et al. 2021; Song et al. 2007), their acquisition may also rely on distinct neural networks (Peigneux et al. 2001; Walker and Stickgold 2005).

The SRTT performance can be assessed via accuracy or response time (RT) measures. These two measures presumably reflect different processes. In general, RT indicates automatic, habitual processes, while accuracy might reflect more controlled, goal-directed processes (Keramati et al. 2011). Participants can complete the SRTT with high accuracy rates throughout the task and most studies report RT-based rather than accuracy-based results. Due to these reasons, we will refer to RT-based results when discussing enhanced or weakened performance in the context of the behavioral effects of rTMS, unless stated otherwise.

### 1.2. Different rTMS Protocols

**Figure 1:**
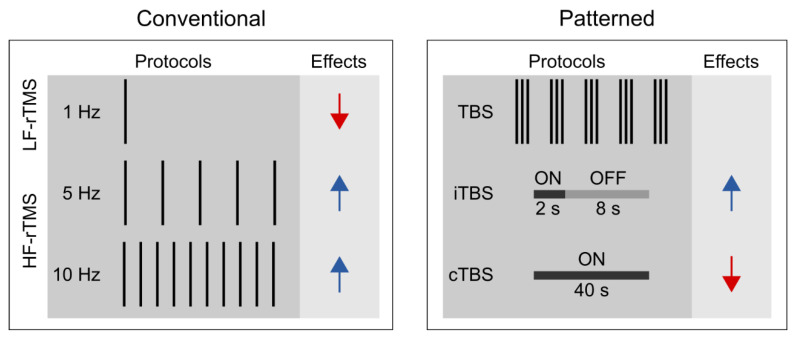
Conventional and patterned rTMS protocols and their assumed aftereffects on corticospinal excitability in M1 based on MEP measurements. Black bars represent the number of delivered rTMS pulses in one second. Red arrows indicate a decrease, whereas blue arrows indicate an increase in corticospinal excitability. Abbreviations: TBS: theta-burst stimulation; cTBS: continuous TBS; iTBS: intermittent TBS; HF-rTMS: high-frequency rTMS; LF-rTMS: low-frequency rTMS.

Unlike single-pulse TMS, rTMS can exert lasting aftereffects on cortical excitability (Fitzgerald et al. 2006; Huang et al. 2017; Klomjai et al. 2015); thus, it is widely used in studying the functional relevance of the targeted brain areas of various cognitive processes (Ferrari et al. 2018; Kim et al. 2004; Ruitenberg et al. 2014; Smalle et al. 2017; Verwey et al. 2002). One can distinguish between conventional and patterned rTMS protocols (see Figure 1). Conventional rTMS protocols use a single frequency (1 Hz, 5 Hz, 10 Hz, etc.) and the temporal pattern of the pulse sequence (i.e., the number of pulses per burst, inter-burst interval, total number of pulses) can vary substantially across studies. For example, one study may deliver 1 Hz rTMS as a single, uninterrupted train of 900 pulses (Bagnato et al. 2005), whereas another one may deliver 300 pulses per burst three times with 60-s-long inter-burst intervals (Chouinard et al. 2005).

Patterned rTMS protocols, such as theta-burst stimulation (TBS) standardize many of the stimulation parameters (Huang et al. 2005). TBS protocols consist of bursts that comprise three pulses at 50 Hz that are repeated at 5 Hz. The most common TBS protocol delivers 600 pulses in total, although shorter (i.e., 300 pulses) or longer (i.e., 1800 pulses) variants also exist (Gentner et al. 2008; McCalley et al. 2021). Based on the presence or absence of intermittent stimulation-free periods, we further distinguish between continuous TBS (cTBS) and intermittent TBS (iTBS) (Huang et al. 2005). In iTBS, the 2-s-long bursts are interrupted by 8-s-long stimulation-free periods, whereas in cTBS, the bursts are delivered continuously.

Based on the induced direction of change in the corticospinal excitability, rTMS is commonly classified according to the frequency of stimulation. Low-frequency rTMS (≤1 Hz) tends to decrease whereas high-frequency rTMS (≥5 Hz) to increase corticospinal excitability (Dayan et al. 2013; Huang et al. 2017; Polanía et al. 2018). Moreover, the effect of rTMS depends on several other stimulation parameters, e.g., the stimulation intensity, the number of delivered pulses, the coil orientation, and the temporal pattern of the protocol (Lang et al. 2006; Pell et al. 2011). It is generally accepted that cTBS tends to decrease whereas iTBS may increase the corticospinal excitability level (Cárdenas-Morales et al. 2010; Hamada et al. 2013; Huang et al. 2005). Still, the proposal that motor cortical low-frequency rTMS and cTBS decrease, whereas high-frequency rTMS and iTBS increase, the corticospinal excitability level, reflects an oversimplified view for the following reasons. First, previous studies have shown substantial intra- and inter-individual variability in TBS effects on cortical and corticospinal excitability (Hamada et al. 2013; Ozdemir et al. 2021). This reproducibility issue may be preferably addressed in future pre-registered multi-center large-scale and high-powered studies (Boayue et al. 2020). Second, the physiological effects of rTMS assessed by fMRI are only incompletely consistent with those suggested by motor cortex studies (Beynel et al. 2020). Specifically, recent meta-analyses suggest that low-frequency rTMS and cTBS increase rather than decrease the resting-state functional connectivity (Beynel et al. 2020; Ji et al. 2017). Finally, the induced aftereffects appear to be polysynaptic, i.e., the locally induced effects can spread to distant brain regions and networks (Beynel et al. 2020). This latter observation can have particular relevance when interpreting the behavioral effects of rTMS in higher cognitive functions, such as visuomotor sequence learning.

## 2. Literature Search and Study Selection

**Figure 2:**
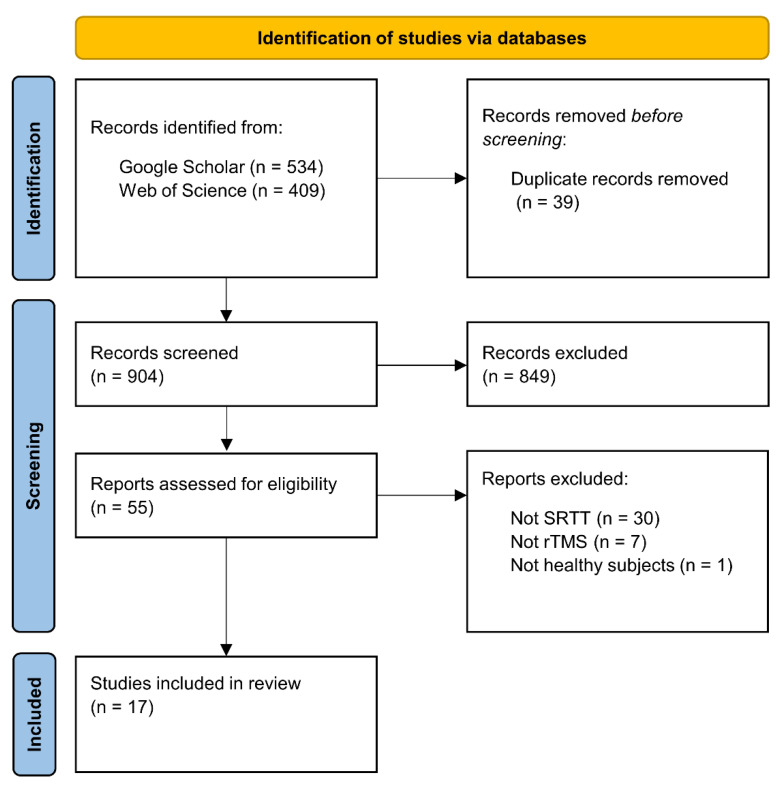
The flowchart of the literature search.

We used the Google Scholar and Web of Science databases for the literature search, which was closed on 1 June 2022. We used the following search syntax: (‘sequence learning’ OR ‘statistical learning’ OR ‘procedural learning’ OR ‘procedural knowledge’ OR ‘implicit learning’ OR ‘implicit memory’ OR ‘motor learning’ OR ‘motor skill’ OR ‘serial reaction time task’) AND (‘TMS’ OR ‘rTMS’ OR ‘TBS’ OR ‘cTBS’ OR ‘iTBS’). This search syntax returned 943 hits on the two databases. Following the PRISMA guidelines (Page et al. 2021), duplicate records were removed before screening the records (39 records). These remaining records were reviewed based on their titles and abstracts and we selected 55 reports that seemed relevant, irrespective of publication year or status (see Figure 2).

The inclusion criterion was the use of rTMS together with the SRTT on healthy human participants. Thirty-eight of the 55 articles were excluded: 30 of them because they used another sequence learning paradigm (e.g., finger tapping task, continuous tracking task); seven of them because they used another NIBS technique (e.g., paired associative stimulation, transcranial direct current stimulation); and one study because it examined the effect of rTMS on the SRTT performance of patients with major depressive disorder. Finally, a total of 17 studies were selected for this review.

## 3. What Factors Determine the Effect of rTMS on Sequence Learning?

Since the methodology of both the SRTT and rTMS is highly diverse, the critical evaluation of the studies was based on the following parameters: (1) stimulated brain areas, (2) rTMS protocols (e.g., ‘inhibitory’ or ‘facilitatory’), (3) stimulated hemisphere, (4) timing of the stimulation, (5) SRTT sequence properties, and (6) other methodological features (e.g., study design).

### 3.1. Stimulated Brain Regions

**Figure 3:**
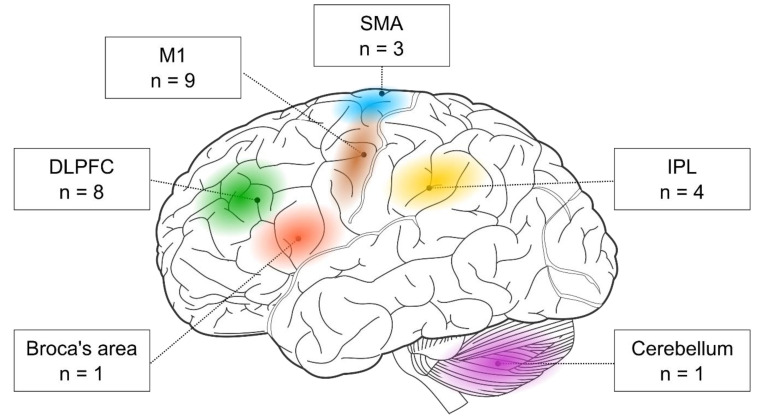
The cortical targets of rTMS-SRTT studies. Brain image was adapted from Hugh Guiney (https://commons.wikimedia.org/wiki/File:Human-brain.SVG; CC BY-SA 3.0; accessed on 25 July 2021).

First, we examined the stimulated brain regions and found that the two most frequent targets were M1 (nine out of 17 studies) and the DLPFC (eight out of 17 studies). In addition, three studies targeted the SMA and four the parietal cortex. One study stimulated the Broca area, and another one the cerebellum (see Figure 3). Below, we evaluate the behavioral effects of rTMS in each cortical target separately. Most of the below-discussed brain areas seem to support visuomotor sequence learning. However, the role of the DLPFC seems more complex since its activity could have a detrimental impact on visuomotor sequence learning.

***M1.*** The role of M1 in motor consolidation goes beyond sequence learning as it engages in the early motor consolidation of elementary motor behavior (Buetefisch et al. 2015; Bütefisch et al. 2004; Muellbacher et al. 2002). The popularity of M1 as a stimulation target is attributed to its implicated role in the initial encoding of sequences and the early consolidation of already learned sequences (Seidler et al. 2005). In the reviewed rTMS studies, typically, low-frequency rTMS or cTBS was applied over M1, resulting in the weakening of the learning process (Clark et al. 2019; Rosenthal et al. 2009; Steel et al. 2016; Wilkinson et al. 2015), or the prevention of offline improvements (Breton and Robertson 2017; Robertson et al. 2005). Interestingly, two studies found an increase in visuomotor skills following low-frequency rTMS or cTBS over M1. In one of them, low-frequency rTMS affected the SRTT indirectly through the prevention of interference with a declarative task (Cohen and Robertson 2011). In the other study, cTBS abolished the decrease in corticospinal excitability, which allowed for offline improvements on an explicit SRTT (Tunovic et al. 2014). Only one of the nine studies attempted to use iTBS over M1, but it did not find any effect on implicit sequence learning (Wilkinson et al. 2010).

***DLPFC.*** The DLPFC has traditionally been identified as a brain area supporting executive functions and working memory (Miller and Cohen 2001; Yuan and Raz 2014). Plasticity changes in the DLPFC seem to be associated with sequence learning (Cao et al. 2022). Lesion studies—where patients with prefrontal lesions show decreased sequence learning on the SRTT (Beldarrain et al. 1999, 2002)—highlight its importance in visuomotor sequence learning, too. However, its functional role is still controversial (Janacsek and Nemeth 2013, 2015). Based on recent models, the DLPFC may act as a neural switch between competitive memory processes (Ambrus et al. 2020; Daw et al. 2005; Lee et al. 2014). On the one hand, it may favor declarative learning and memory (e.g., memory for events and facts), as well as top-down processes. However, if the situation requires acquiring new regularities (e.g., a completely new pattern or sequence), it recedes. A potential mediator role of the DLPFC is also supported by the findings of a TBS study, where the learning of linguistic sequences was enhanced due to the disruptive stimulation of the DLPFC (Smalle et al. 2022). Out of the eight identified studies, three studies found that rTMS over the DLPFC weakened implicit sequence learning (Cohen and Robertson 2011; Pascual-Leone et al. 1996; Robertson et al. 2001). In one of them, DLPFC stimulation reduced learning on the SRTT indirectly by interfering with a declarative task (Cohen and Robertson 2011). Examining explicit sequence learning, two of the six studies found an enhancement for cTBS over the DLPFC (Galea et al. 2010; Tunovic et al. 2014). However, one recent study found no effect of DLPFC stimulation on explicit the SRTT (Gann et al. 2021). Two additional studies used probabilistic instead of deterministic sequences. One of them found that low-frequency rTMS over the DLPFC led to better performance on this sequence type (Ambrus et al. 2020), while the other study found no effect of DLPFC stimulation on performance (Wilkinson et al. 2010).

***SMA.*** Some neuroimaging studies reveal that, besides M1, another motor area, the SMA also appears to be involved during the SRTT (Hazeltine et al. 1997; Seidler et al. 2005). According to an fMRI study, SMA activation is associated with the performance of sequential movements (Hikosaka et al. 1996). Additionally, a PET study suggests that the SMA is involved in the execution of previously learned sequences rather than in the acquisition of sequences (Honda et al. 1998). In more recent studies, the SMA was found to be involved in the automatization of sequential movements (Shimizu et al. 2020) and the consolidation of implicit sequence knowledge (Verwey et al. 2022). We found that only three studies have targeted the SMA. Two of them found no association between SMA stimulation and learning on the SRTT (Pascual-Leone et al. 1996; Wilkinson et al. 2010). The third study supported the role of the SMA in the intermanual transfer of the sequence (Perez et al. 2008), which refers to the phenomenon in which the procedural knowledge acquired by one hand can be performed by the other hand (Grafton et al. 2002; Japikse et al. 2003). The functional role of the SMA in visuomotor sequence learning should be confirmed by administering rTMS at the appropriate learning stage.

***Parietal cortex.*** According to previous behavioral studies, a motor response is not strictly necessary for the acquisition of complex sequences; monitoring in itself can lead to learning (Nemeth et al. 2009; Song et al. 2008; Zolnai et al. 2022). Based on functional neuroimaging studies, the inferior parietal lobule (IPL) encodes the sequence at a general, abstract level, independently of the response mode (Grafton et al. 1998; Hikosaka et al. 1999). We found four studies that aimed to investigate the effect of rTMS over the parietal cortex on sequence learning. One study verified a crucial contribution of this area to perceptual sequence learning because the application of cTBS over the IPL resulted in the prevention of learning on a probabilistic SRTT (Rosenthal et al. 2009). According to the work of Breton and Robertson (2017), the IPL seems to play a significant role in the consolidation of sequence knowledge as well, because low-frequency rTMS blocked offline improvements on an implicit SRTT (Breton and Robertson 2017). In contrast, two of the four reviewed studies found no effect of stimulation of the IPL on sequence learning; therefore, its role remains elusive.

***Broca’s area.*** In the field of sequence learning, Broca’s area has been primarily tested on artificial grammar learning tasks (De Vries et al. 2010; Uddén et al. 2017), where participants need to extract rules from artificially generated grammatical sequences (Reber 1967, 1989). Because the acquisition of the grammar of a language is connected to sequence learning (Nemeth et al. 2011), it is an interesting question whether this brain area is also involved in the acquisition of non-linguistic visuomotor sequences. Only one study has examined the role of Broca’s area and showed that cTBS over the BA 44 prevented the learning on an implicit SRTT (Clerget et al. 2012).

***Cerebellum.*** The role of the cerebellum in sequence learning is highly uncertain in the literature (Baetens et al. 2020; Janacsek et al. 2020). While the detrimental effect of cerebellar damage on sequence learning assumes its essential role (Dirnberger et al. 2013; Doyon et al. 1997; Gomez-Beldarrain et al. 1998; Shin and Ivry 2003), neuroimaging studies do not always support this hypothesis (Janacsek et al. 2020; Kóbor et al. 2022; Seidler et al. 2002; van der Graaf et al. 2006). However, a recent fMRI study revealed that the cerebellum is involved in the early phase of task performance and coordination since its activity diminishes as the task becomes well practiced (Hermsdorf et al. 2020). On the other hand, the functional role of the cerebellum has been mainly studied in motor adaptation tasks (Doppelmayr et al. 2016; Galea et al. 2011; Jayaram et al. 2012). Only one study investigated the causal role of the cerebellum in visuomotor sequence learning and showed that low-frequency rTMS over the lateral cerebellum resulted in a significant weakening in sequence learning (Torriero et al. 2004).

### 3.2. ‘Inhibitory’ and ‘Facilitatory’ rTMS Protocols

It is common to assume a linear relationship between the direction of the produced aftereffects on cortical excitability and the behavioral effects of rTMS. According to this view, low-frequency rTMS and cTBS (i.e., the ‘inhibitory’ protocols) might induce functional inhibition/disruption, whereas high-frequency rTMS and iTBS (i.e., the ‘excitatory’ protocols) might lead to functional improvements/enhancement. Although this generally accepted dichotomy between the stimulation frequency and the direction of the produced cognitive aftereffects is likely oversimplified, several studies discuss the results in this framework.

In the following, we delineate several reasons that it is challenging to predict the functional aftereffects of rTMS solely based on the protocol type (i.e., ‘inhibitory’ or ‘excitatory’). First, there is substantial interindividual variability when inducing corticospinal excitability changes in M1. While group-level data might show frequency-dependent modulatory effects, they can vary significantly across individuals (Hamada et al. 2013; Maeda et al. 2000), and even within individuals (Goldsworthy et al. 2021). Biological (e.g., age, time of the day, genetics, brain state) and methodological factors (e.g., stimulation parameters, measures for the effect) both may be responsible for the intra- and interindividual variability of the rTMS effect (Huang et al. 2017). One possible strategy to decrease variability effects is increasing the specificity of the stimulation. This can be achieved by a novel rTMS technique, called quadripulse stimulation (QPS), that uses repetitive monophasic pulses, instead of biphasic pulses resulting in smaller variability in the after-effects (Simeoni et al. 2016; Tiksnadi et al. 2020).

Second, it is unclear whether a given protocol that may decrease the cortical excitability in M1 produces the same physiological effects in other cortical areas. For instance, some authors speculate that there might be overlaps in the produced aftereffects at least within the frontal cortex (e.g., M1 and DLPFC, as discussed in de Jesus et al. 2014). Third, this view may miss the brain’s endogenous and dynamic compensatory mechanisms to external perturbations. For example, due to the interhemispheric compensation, decreasing the excitability level of the left DLPFC with low-frequency rTMS may lead to the compensatory recruitment of the right DLPFC (Ambrus et al. 2020). Fourth, the stimulation frequency is only one of many crucial stimulation parameters that can shape the direction of aftereffects. For example, the facilitating effect of high-frequency rTMS requires inter-train intervals; otherwise, it is more likely to produce an inhibitory effect (Rothkegel et al. 2010).

Based on these arguments, it is conceivable to expect that the behavioral effects of rTMS may not always match the alterations in cortical excitability. Consequently, ‘facilitatory’ protocols may not always enhance, and ‘inhibitory’ protocols may not necessarily weaken the performance. However, due to the prevailing use of these terms in the literature, we tentatively evaluated the link between the protocol type and the direction of the induced behavioral effects.

In the present review, 16 out of the 17 articles found an rTMS effect on sequence learning, of which 12 studies followed the pattern that is intuitively expected based on the ‘inhibitory—facilitatory’ dichotomy. These studies found that ‘inhibitory’ protocols (i.e., low-frequency rTMS, cTBS) indeed weakened the performance on the SRTT. However, three studies found that the ‘inhibitory’ stimulation of the DLPFC led to enhanced performance on the SRTT (Ambrus et al. 2020; Galea et al. 2010; Tunovic et al. 2014). These findings may be explained by the mediating role of the DLPFC between competitive memory processes (Ambrus et al. 2020; Daw et al. 2005). Alternatively, the behavioral effects of rTMS may be due to changes in the cortical excitability of another brain area that is, however, part of the same neural network. For example, Cao et al. (2018) found that ‘facilitatory’ iTBS over the DLPFC decreased, while ‘inhibitory’ cTBS over the DLPFC increased the cortical excitability in M1 (Cao et al. 2018). In another study, rTMS affected SRTT performance indirectly by preventing interference with a declarative task (Cohen and Robertson 2011).

Out of the 17 reviewed studies, only three applied ‘facilitatory’ protocols, and only one revealed its effect on sequence learning (Pascual-Leone et al. 1996). This study showed that 5 Hz rTMS (Pascual-Leone et al. 1996) weakened the performance on the SRTT. However, the other two studies found no effects of rTMS (Gann et al. 2021; Wilkinson et al. 2010). Therefore, the evidence on the impact of ‘facilitatory’ protocols (≥5 Hz and iTBS) on the SRTT is still lacking and requires further exploration.

### 3.3. Stimulated Hemisphere(s)

Many studies have used rTMS to better understand the hemispheric involvement of a given brain region when performing the SRTT. These studies typically ask whether the left or right brain area (e.g., M1) is causally involved in a specific task phase (e.g., learning phase). To this aim, most studies have stimulated the left or the right hemisphere at a time and studied whether rTMS could modulate the performance.

Performing the SRTT may require using only one hand or both hands. When the participants perform the SRTT with only one hand (e.g., the right hand), the stimulation may target the contralateral (i.e., left) or ipsilateral (i.e., right) hemisphere. Eleven of the 17 studies targeted the contralateral hemisphere (see Table 1). In studies where only the left hemisphere was stimulated, only right-handed participants were included (see Table 1).

However, studies targeting the dominant hemisphere (based on M1) may neglect the possibility that the non-stimulated hemisphere can take over the function of the stimulated one (Andoh and Martinot 2008; Sack et al. 2005), potentially influencing the results. Applying sequential bilateral stimulations (i.e., delivering the same rTMS protocol over a given cortical target consecutively on each hemisphere) may be a promising solution to overcome the possible interhemispheric compensatory mechanisms (Ambrus et al. 2020).

Considering the side of the stimulation, some studies have targeted both hemispheres in separate experimental groups. Using this method, Galea et al. (2010) successfully demonstrated that cTBS over the right DLPFC improved visuomotor sequence learning to a greater extent than the left DLPFC (Galea et al. 2010). Another study revealed a dissociation between cerebellar hemispheres: the stimulation of the right cerebellar hemisphere weakened sequence learning regardless of which hand was used, while the interference with the left cerebellar hemisphere affected only through the ipsilateral hand (Torriero et al. 2004). Therefore, this method is suitable for exploring potential lateralization effects as well.

For non-motor brain areas, it is worth targeting both hemispheres separately and applying sequential bilateral protocols. This approach can avoid hemispheric compensatory mechanisms and reveal the possible dissociation between hemispheres (Ambrus et al. 2020). Furthermore, the sequential bilateral stimulation for the two-handed version of the task may be a particularly good solution.

### 3.4. Timing of the Stimulation

Motor memory traces can be strengthened in two ways: when performance is gradually improving during practice (online learning) and when performance improves between two training sessions without any practice (offline learning) (Cohen et al. 2005; Robertson et al. 2004). Sequence learning is a multi-stage process: it consists of the learning, consolidation, and retrieval phases. Different brain regions and neural networks may be recruited at each learning stage (Veldman et al. 2018); therefore, we evaluated the studies in each stage separately.

**Figure 4:**
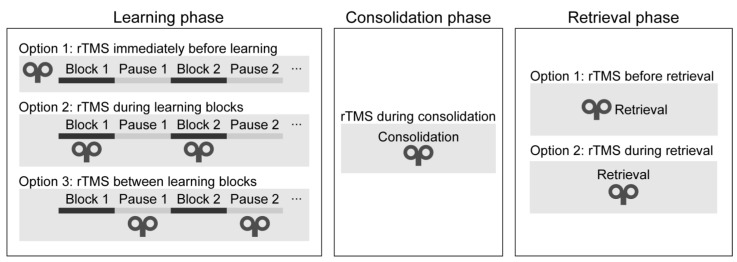
Timing of the stimulation in each task phase.

***Learning phase.*** One can deliver rTMS immediately before or during the learning phase (see Figure 4). During the learning phase, rTMS may be applied simultaneously with the task performance or between the learning blocks. Only two studies have applied rTMS during the initial sequence learning process (so-called ‘online stimulation’). In one study, high-frequency rTMS over the DLPFC led to a performance decrease on the SRTT (Pascual-Leone et al. 1996). In a more recent study, the authors applied low-frequency rTMS over the DLPFC between the learning blocks and found a performance improvement on an alternating SRTT (Ambrus et al. 2020). Most research delivered stimulation immediately before task performance (so-called ‘offline stimulation’; see Table 1). Most of them have found decreased learning of visuomotor sequence learning (Clark et al. 2019; Clerget et al. 2012; Torriero et al. 2004; Wilkinson et al. 2015).

***Consolidation phase.*** One may apply rTMS after the learning phase to verify its effect on memory consolidation (see Figure 4). In one study, cTBS over the DLPFC improved performance after an 8-h-long offline period (Galea et al. 2010). In contrast, low-frequency rTMS over M1 blocked offline improvements on an implicit SRTT over the day (Robertson et al. 2005), as well as on an explicit SRTT after sleep (Breton and Robertson 2017). We conclude that rTMS over the DLPFC and M1 can influence the development of memory traces because their stimulation leads to changes in the consolidation process.

***Retrieval phase.*** Finally, rTMS may be applied immediately before or during the recall phase. To the best of our knowledge, no studies have applied rTMS immediately before or during the recall phase as of the day of the literature search. Thus, it is unclear whether rTMS can modulate the recall of well-acquired sequence knowledge.

### 3.5. Type of the SRTT Sequence

The effect of stimulation may depend on the SRTT sequence type. Several new versions of the SRTT have emerged that can differ in three crucial dimensions: the sequence applied can be (1) implicit or explicit, (2) deterministic or probabilistic, and (3) first-order conditional (FOC) or second-order conditional (SOC) (as defined in section *Different variations of the SRTT*).

***Implicit* vs. *explicit sequences.*** The most commonly used version of the SRTT uses implicit sequences (here, 13 out of the 17 reviewed studies used an implicit SRTT). However, its explicit version (i.e., the existence of the predetermined sequence is revealed to the participants before learning) can also be applied if the goal is to test intentional learning or declarative knowledge of the sequence. Among the reviewed rTMS articles, we found that rTMS could affect explicit and implicit learning (Table 1). However, the study of Breton and Robertson (2017) showed evidence that the degree of sequence awareness can influence the effect of rTMS. They examined the role of M1 and the IPL in both the implicit and explicit SRTT and revealed a double dissociation: low-frequency rTMS over the IPL prevented offline improvement in the implicit but not in the explicit task. On the other hand, the same stimulation over M1 prevented offline improvement in the explicit but not in the implicit task (Breton and Robertson 2017).

***Deterministic* vs. *probabilistic sequences.*** In the classic SRTT, stimuli follow a fixed order, creating a deterministic sequence (Nissen and Bullemer 1987; Shanks 2005). Probabilistic types of the SRTT also exist, where the sequence is hidden in noise; therefore, learning is more likely to remain implicit (Howard et al. 2004; Song et al. 2007). Five out of 17 studies used probabilistic sequences, and 12 used deterministic sequences (Table 1). All of the studies employing probabilistic sequence learning tasks found behavioral effects of rTMS. In four studies, learning deteriorated (Rosenthal et al. 2009; Steel et al. 2016; Wilkinson et al. 2010, 2015), and, in one study, learning was improved (Ambrus et al. 2020). On the other hand, deterministic sequence learning performance was successfully manipulated in 11 out of the 12 studies: in eight studies, learning was disrupted (Breton and Robertson 2017; Clark et al. 2019; Clerget et al. 2012; Pascual-Leone et al. 1996; Perez et al. 2008; Robertson et al. 2005, 2001; Torriero et al. 2004), and, in three studies, learning was improved (Cohen and Robertson 2011; Galea et al. 2010; Tunovic et al. 2014). Therefore, it seems that rTMS can equally modify the learning of both probabilistic and deterministic sequences. However, we cannot draw firm conclusions due to the small number of studies with probabilistic sequences.

***FOC* vs. *SOC sequences.*** Another critical factor is the statistical structure of the sequence. In the simpler first-order conditional (FOC) sequences, elements can be predicted by the preceding one. On the other hand, in the more complex second-order conditional (SOC) sequences, it is the combination of two consecutive elements that predicts the forthcoming one. Clark et al. (2019) investigated the role of M1 in the acquisition of simpler FOC and more complex SOC sequences. According to their findings, cTBS over M1 resulted in poorer learning of the FOC sequence compared to the SOC sequence. These findings support the hypothesis that the acquisition of FOC and SOC sequences may rely on different neural networks: simpler FOC sequences are processed by a circuitry involving M1, while more complex SOC sequences are associated with an expanded network, including Brodmann area 44 (BA44) and the DLPFC (Ashe et al. 2006; Lum et al. 2018). Although working with discrete sequence production task instead of the SRTT, an rTMS study also revealed the distinct role of the pre-SMA in more complex sequences compared to simpler ones (Ruitenberg et al. 2014). Based on these promising results, future studies may investigate FOC and SOC sequences targeting non-motor areas too.

### 3.6. Methodological Features

Next, we summarize some of the key methodological aspects of rTMS studies, focusing on the research design, the type of control, the localization techniques of target areas, the determination of the stimulation intensity, and the interpretation of different outcome measures (see Table 2 and Table 3).

***Research design and the type of control condition.*** The reviewed rTMS studies mostly applied between-subjects research designs, and it was less common to use within-subjects or mixed study designs (see Table 2). Typically, the stimulation group is compared to a control group that goes through the same procedure except for the stimulation parameters. A frequent study design is when the control group receives sham stimulation (i.e., using a placebo coil or tilting the coil away from the skull to a certain degree) where they can hear the clicking sound as the stimulation group, but the intensity of the TMS-induced intracranial electric field is negligible. One known limitation of this approach is that the rTMS-induced perceptual adverse effects (e.g., somatosensory sensations—in part—due to cranial muscle contractions) are not fully comparable to the verum stimulation. Therefore, the blinding of the participants might not be maintained effectively (Duecker and Sack 2015).

Instead of the sham condition, studies may apply an active control condition. Here, the stimulation is delivered over a ‘control’ site while using an identical stimulation protocol as for the main cortical area. This option has the disadvantage that the brain area chosen as the control site might play a role in the tested function because the underlying brain networks involved in the given task might not be fully revealed yet.

In the reviewed studies, sham stimulation was the most commonly chosen approach—here, ten studies used it as a control. On the other hand, three studies chose active control with different control sites (i.e., vertex, occipital, and parietal sites). In two studies, members of the control group were simply not exposed to rTMS (Pascual-Leone et al. 1996; Torriero et al. 2004), one study compared cTBS to intermediate TBS (Tunovic et al. 2014), and another study only compared two active stimulation protocols (cTBS and iTBS) to each other (Gann et al. 2021) (see Table 2).

***Localization technique.*** The different methods of target area localization may also contribute to the inconsistency of results (De Witte et al. 2018; Sack et al. 2009). The target area for M1 is traditionally identified during the motor threshold measurement (see Table 3). In the reviewed studies, non-motor cortical regions were mostly located by MRI-guided neuronavigation systems, typically using individual MRI recordings. Most of them successfully demonstrated the causal role of the given regions of the sequence learning process (Ambrus et al. 2020; Clerget et al. 2012; Galea et al. 2010; Rosenthal et al. 2009). Wilkinson et al. (2010) localized their non-motor target areas with the 10–20 EEG system or by the 5-cm rule with M1 as a reference interestingly, they did not find any effect. As the DLPFC is an extensive area, different localization techniques lead to slightly different cortical targets (e.g., BA9, BA46). According to an fMRI investigation, BA9 shows greater activity during sequence learning than BA46 in young adults (Aizenstein et al. 2006). Therefore, localization may be an important parameter, and future studies should preferably use neuronavigation to precisely target and document the coil parameters concerning individual gyral folding patterns. Moreover, only two of the 17 studies applied fMRI to study the hemodynamic effects of rTMS (Gann et al. 2021; Steel et al. 2016). A combination of NIBS and neuroimaging techniques is warranted to gain a clearer picture of what exactly happens in the brain and how this leads to better or poorer sequence learning performance.

***Stimulation intensity and the total number of pulses.*** The overwhelming majority of rTMS studies determine the stimulation intensity based on the motor threshold (Turi et al. 2021). In line with this, 16 of the 17 studies used the motor threshold; however, the stimulation intensities varied substantially (see Table 3). Only one study used a fixed intensity (Ambrus et al. 2020); however, it successfully modulated the SRTT performance. Although adjusting the intensity to the motor threshold is a reasonable method for M1 stimulation, the physiological justification for this procedure is less clear for non-motor cortical areas. Moreover, the high diversity of the chosen intensities might make it even more challenging to compare the results of different studies.

In addition to the stimulation intensity, the total number of pulses can also be decisive for the effectiveness of rTMS (Thut and Pascual-Leone 2010). Specific stimulation protocols may standardize the total number of pulses. In 12 out of the 17 reviewed articles, the number of delivered pulses was 600. Three studies used more (i.e., 900, 1200, and 3000), whereas one study used fewer pulses (i.e., 300; see Table 3). With both a larger and smaller delivered total number of pulses, a significant effect of stimulation was demonstrated.

## 4. Discussion

In this review, we sought to provide insight for future rTMS studies into the factors that might affect its efficiency in modulating visuomotor sequence learning and the behavioral outcome of the SRTT. It has been found that visuomotor sequence learning as measured by the SRTT was successfully altered by rTMS in most of the reviewed studies. Furthermore, we identified and examined the most critical factors that govern the behavioral effects of rTMS on visuomotor sequence learning as measured by the SRTT. These factors include rTMS parameters (e.g., frequency, timing, etc.) and task characteristics (e.g., sequence type). Moreover, the reviewed studies were scrutinized based on additional methodological details (e.g., study design, and localization technique).

We found that the two most frequently stimulated cortical targets were M1 and the DLPFC, and only a few studies have targeted the parietal, supplemental motor, and cerebellar areas. Low-frequency rTMS and cTBS (i.e., ‘inhibitory’ protocols) over M1 typically weakened sequence learning performance. Several of the reviewed studies proved the functional role of M1 in offline improvements or the consolidation of the acquired sequence knowledge (Breton and Robertson 2017; Robertson et al. 2005; Tunovic et al. 2014). However, a related rTMS study using a finger tapping task instead of the SRTT found that M1’s role is limited to the early post-training period and is abolished in the delayed stages of consolidation (Hotermans et al. 2008). Accordingly, the exact role of M1 in the dynamics of sequence knowledge consolidation needs to be further clarified in the future. On the one hand, it is suggested to examine the functional role of the M1 with rTMS in each learning phase (see Figure 4). On the other hand, according to longitudinal fMRI studies, M1 appears to be irrelevant in human motor sequence learning, while there are targets with greater potential (e.g., SMA, parietal regions) to modify sequence learning (Berlot et al. 2020; Yokoi et al. 2018). Thus, it is also suggested to place a greater emphasis on these less examined brain regions.

The results are even less consistent for the DLPFC. In some early studies, low-frequency rTMS and cTBS over the DLPFC weakened learning (Pascual-Leone et al. 1996; Robertson et al. 2001). However, more recent studies have found no effects (Gann et al. 2021; Wilkinson et al. 2010) or even enhanced learning (Ambrus et al. 2020; Galea et al. 2010). One possible explanation for the behavioral improvement might lie in the competitive relationship between distinct memory systems supporting human learning: model-based and model-free learning processes (Daw et al. 2005; Nemeth et al. 2013; Smalle and Möttönen 2023; Smittenaar et al. 2013). An antagonistic relationship can be witnessed between the two systems—a decrease in one can lead to the prominence of the other, and vice versa (Janacsek and Nemeth 2022; Pedraza et al. 2023). Beyond visuomotor sequence learning, the disruptive stimulation of the DLPFC enhanced the learning of linguistic sequences as well (Smalle et al. 2017, 2022). We hypothesize that the DLPFC might play a mediating role between these two learning processes. Since its activity favors model-based processes, sequence learning, which is at the other end of the scale, declines. However, applying low-frequency rTMS may promote these model-free, associative learning processes via its implicated inhibitory effects on the DLPFC (Ambrus et al. 2020; Daw et al. 2005). Similarly, the cognitive cost hypothesis states that the maturation of the prefrontal cortices (peaks starting from adolescence) supports higher top-down cognitive processes that are required in adult life, but impedes habitual, associative learning processes of the model-free system (Janacsek et al. 2012; Juhasz et al. 2019; Smalle and Möttönen 2023; Tóth-Fáber et al. 2023). Another speculative hypothesis in alignment with this line of thinking posits that stimulating the DLPFC reduces the influence of prior knowledge, thereby promoting learning that leans towards model-free learning based on weak priors. Consequently, 1 Hz rTMS stimulation inhibits access to long-term models, thereby enhancing learning when new patterns or sequences need to be acquired, as top-down processes play a lesser role in such scenarios (Ambrus et al. 2020).

The less consistent findings observed over the DLPFC might be partially explained by the differences in the localization techniques. The DLPFC is an extensive functional brain structure and there are several methods to target it. We identified eight studies that targeted the DLPFC and three of them used scalp-based heuristics (i.e., 5-cm rule). Three of the remaining studies used coordinates for neuronavigation that they modified from previous neuroimaging studies (Cohen and Robertson 2011; Galea et al. 2010; Tunovic et al. 2014), whereas one study applied the exact coordinates from a previous meta-analysis (Ambrus et al. 2020). The last study used individual, resting-state functional connectivity-derived coordinates to target the DLPFC (Gann et al. 2021). In contrast, all studies that targeted M1 used, in general, the same method by stimulating the motor ‘hot spot’ of a hand muscle. Taken together, the identified methods likely targeted different parts of the DLPFC and, in turn, different functional brain networks (Cardenas et al. 2022). Individualized functional brain network-based rTMS targeting is an interesting future avenue to understand better the neuronal networks crucial for visuomotor sequence learning (Cash et al. 2021; Gann et al. 2021; Lynch et al. 2022). To sum up, our findings suggest that stimulating M1 or the DLPFC could modulate sequence learning; however, the underlying mechanisms and the reasons behind the discrepancies between studies should be clarified in the future. To do so, re-evaluating existing findings over establishing new research questions would be welcomed. Moreover, the systematic examination of these potential target areas’ functional roles in all learning phases within one study design would be an enormous step toward the understanding of the exact underlying neural mechanisms.

Furthermore, it should be emphasized that it is not solely the functioning of local brain areas but rather the connectivity within expansive neural networks that is responsible for complex cognitive functions such as visuomotor sequence learning. In a recent neuroimaging study, implicit visuomotor sequence learning was associated with weakened functional connectivity between the superior frontal gyrus and the brain networks involved in top-down control processes (e.g., dorsal attention and language networks) (Park et al. 2022). In another recent study, TBS disrupted motor memory consolidation on a finger tapping task by modulating hippocampal and striatal networks via prefrontal stimulation (Gann et al. 2023). Gann and colleagues’ work highlights the possibility of reaching the deep brain structures beside cortical targets with rTMS through spreading activation. Thus, studies should focus on the local as well as network effects of rTMS to modulate visuomotor sequence learning. Only two of the reviewed studies examined the effect of rTMS on the SRTT using fMRI. Although these studies did not find any behavioral effect of rTMS on the SRTT, they supported the view that rTMS does not induce local or distant changes in the activity of individual brain regions; instead, it changes the functional connectivity patterns between neural networks. To capture the interchangeable effects of neural and behavioral changes in rTMS and the SRTT, the inclusion of neuroimaging techniques is warranted.

We found that most studies used ‘inhibitory’ rTMS protocols with the aim to reduce the cortical excitability level of the targeted brain areas and weaken sequence learning (Breton and Robertson 2017; Clark et al. 2019; Clerget et al. 2012; Perez et al. 2008; Rosenthal et al. 2009; Steel et al. 2016; Torriero et al. 2004; Wilkinson et al. 2015). In contrast, only a handful of studies used ‘facilitatory’ rTMS, and they led to null findings (Gann et al. 2021; Wilkinson et al. 2010). One possible explanation for the observed pattern of findings is that it might be more challenging to enhance cognitive performance in healthy participants. Moreover, we should not rule out the potential file-drawer effect because substantially fewer studies published ‘facilitatory’ than ‘inhibitory’ protocols. Nevertheless, studies using high-frequency rTMS and iTBS are warranted to reveal the efficacy of these protocols to modify sequence learning. However, linking the expected physiological effects together with the induced behavioral findings is difficult to interpret due to the following reasons. First, the physiological mechanisms of rTMS over motor and especially over non-motor cortical regions are less clear. Studies report substantial intra- and inter-individual variability in the induced physiological and behavioral effects. Second, we lack a cognitive model of visuomotor sequence learning and consequently, it is difficult to predict the behavioral consequences of a de facto increase/decrease in cortical excitability levels in a given brain region (if this effect is possible to induce locally at all) and related brain networks. Third, these issues may be also explained by homeostatic plastic effects, which are a form of plasticity that is thought to balance the effects of Hebbian (synapse-specific) plasticity to maintain the stability of neuronal networks (Turrigiano 2012). In line with this, previous studies have found that a ‘facilitatory’ protocol may inhibit, while an ‘inhibitory’ protocol may facilitate, task performance on motor sequence learning (Jung and Ziemann 2009; Shimizu et al. 2020). While the notion of homeostatic plasticity may provide a plausible explanation for a limited set of the observed behavioral effects, it has insufficient predictive power to confidentially forecast the direction of behavioral outcomes induced by ‘facilitatory’ and ‘inhibitory’ rTMS protocols. Moreover, the activity of specific brain regions (e.g., DLPFC) might be associated with hindered sequence learning performance. In such cases, the use of ‘inhibitory’ rTMS that interferes with these brain regions could eventually lead to the facilitation of sequence learning.

Interpreting these findings based on the commonly used frequency-based heuristics that have been established over the motor cortex may result in seemingly paradoxical behavioral findings. The sole application of rTMS cannot adequately address these paradoxical findings because the stimulation-induced network effects are still elusive. Therefore, we conclude that developing a cognitive model of visuomotor sequence learning requires the synergic combination of functional neuroimaging and rTMS. In addition, applying ‘facilitatory’ and ‘inhibitory’ rTMS protocols accompanied by a within-subjects study design would result in a clearer understanding of the effect of these different protocols on sequence learning capacities.

One can apply rTMS at different stages of sequence learning and, therefore, systematically study its functional effects. For example, one can deliver rTMS before or during the learning phase. If the research question tackles consolidation processes, one could apply rTMS after the learning phase, and study how the performance develops over the next training session. Similarly, it is possible to deliver rTMS after the consolidation period or immediately before the retrieval of the acquired sequence, which allows the examination of how rTMS interferes with the retrieval. In this review, we found that so far studies have mainly applied rTMS before or during the learning phase or during the consolidation phase (i.e., after the learning phase), yet different studies have used different rTMS protocols. Therefore, in a future research series, it would be desirable to apply the same rTMS protocol systematically at every stage of the visuomotor sequence learning task. Although using a continuous tracking task instead of the SRTT, the findings of two related studies that applied the same rTMS protocols in different stages of the learning process support this suggestion. The effect of both low- and high-frequency rTMS over the dorsal premotor cortex was changed according to whether the stimulation was applied before or after practice (Boyd and Linsdell 2009; Meehan et al. 2013). This example highlights that the proposed approach could help to better understand the functional effects of a given rTMS protocol that may depend on the phase of the task. In the long term, this improved understanding could contribute to developing a unified cognitive model of visuomotor sequence learning.

Our findings also revealed that the stimulation parameters and the task characteristics were highly diverse in the reviewed studies, which could have influenced the results to a great extent. This diversity makes direct comparisons between the studies challenging. Different stimulation protocols (i.e., conventional rTMS vs. TBS), stimulation timings (i.e., before, during, or after learning), control stimulation (i.e., no control, sham stimulation, active control), and stimulation intensities were utilized in almost all studies. Although both repetitive stimulation techniques seem to modulate visuomotor sequence learning effectively, a recent study suggests that the conventional low-frequency rTMS has a greater inhibitory effect on motor sequence learning than cTBS (Glinski 2021; Verwey et al. 2022). Based on the protocol type, stimulation intensity, stimulation frequency, stimulation timing, total number of pulses, and control stimulation, 11 out of the 17 studies used unique protocols. In the remaining six studies, we found two stimulation protocols that were repeatedly used. It was also noted that these two protocols belonged to two research groups. Thus, a total of 13 unique research protocols emerged out of the 17 studies. In addition to answering new questions and testing novel methods, it seems necessary to verify whether the already existing key findings are replicable with the same (i.e., direct replication) or slightly different stimulation parameters (i.e., conceptual replication). Additionally, future studies may explore the potential differences in the efficacy of rTMS and TBS protocols (along with the different stimulation parameters) in modulating visuomotor sequence learning.

Despite the high diversity in most of the methodological features, the study designs of the reviewed articles were relatively uniform. Most of the studies (11 out of the 17) chose a within-subjects design. Six studies used a between-subjects design. While comparing independent groups that receive different stimulation protocols has its advantages (e.g., eliminating practice effect), it does carry drawbacks to consider. For instance, significant individual differences could lead to baseline discrepancies in sequence learning ability between the groups. As a result, facilitatory effects could be masked since the improvement in sequence learning due to the ‘facilitatory’ rTMS protocol is still inferior to that of the control group with stronger sequence learning abilities. Therefore, future studies are encouraged to confidently opt for within-subjects designs, or, alternatively, minimize possible baseline group differences by increasing their sample size.

Here, the most important factors and their parameter spaces that are worth considering when studying visuomotor sequence learning via rTMS were reviewed. Future studies that systematically examine all these factors (e.g., by incorporating all learning phases) within a single study design and with a sufficiently large sample size would be highly valuable. Based on the reviewed studies, we conclude that rTMS could produce exciting behavioral findings when targeting M1 and the DLPFC. However, other less examined yet promising areas like the SMA and parietal areas, as well as deep brain structures, should be further examined. However, we know little about the induced effects on neuronal network levels when stimulating cortical targets separately. Future studies should preferably combine rTMS with electrophysiological and/or neuroimaging methods since the synergic combination of functional neuroimaging with neuromodulation is a necessary step to develop a cognitive model of visuomotor sequence learning. It could facilitate a clearer view of the underlying behavioral and neural effects of rTMS on visuomotor sequence learning and help to overcome the commonly used frequency-based interpretation of rTMS-induced cognitive effects.

## Figures and Tables

**Table 1:** The effect of different rTMS protocols on visuomotor sequence learning according to the target area, sequence type, and timing of the stimulation.

Target Area	Authors and Year	rTMS Protocol	Hemisphere	Timing of Stimulation	Type of Sequence	Outcome
**M1**						
	(Robertson et al. 2005)	1 Hz rTMS	Left only	After learning	12-item implicit deterministic	Blocked offline improvements over the day, but not overnight
	(Breton and Robertson 2017)	1 Hz rTMS	Left only	After learning	12-item implicit/explicit deterministic	Blocked offline improvements in explicit, but not in implicit task
	(Tunovic et al. 2014) (Experiment 3)	cTBS	Right only	After learning	12-item explicit deterministic	Offline improvements after cTBS
	(Cohen and Robertson 2011) (Experiment 2)	1 Hz rTMS	Right only	After learning	12-item implicit deterministic	Increased learning after 12 h consolidation by preventing interference with a declarative task
	(Wilkinson et al. 2010)	cTBS, iTBS	Left only	Before learning	12-item implicit probabilistic	Learning was prevented by cTBS
	(Wilkinson et al. 2015)	cTBS	Left only	Before learning	12-item implicit probabilistic	Decreased initial sequence learning and recall
	(Steel et al. 2016)	cTBS	Left only	Before learning	12-item implicit probabilistic	Learning was disrupted
	(Rosenthal et al. 2009) (Experiment 1, 2)	cTBS	Contralateral to dominant hand	Before learning	12-item implicit probabilistic	Learning was disrupted in manual, but not in perceptual task
	(Clark et al. 2019)	cTBS	Left only	Before learning	12-item implicit deterministic	Decreased learning in simple, but not in a more complex sequence
**DLPFC**						
	(Pascual-Leone et al. 1996)	5 Hz rTMS	Left or right in separate conditions	During learning	12-item implicit deterministic	Learning was disrupted
	(Robertson et al. 2001)	1 Hz rTMS	Contralateral to dominant hand	Before learning	10-item implicit deterministic	Learning was prevented in spatial, but not in color cue guided task
	(Wilkinson et al. 2010)	cTBS	Left only	Before learning	12-item implicit probabilistic	No effect on learning
	(Gann et al. 2021)	cTBS, iTBS	Left only	Before learning	8-item explicit deterministic	No effect on learning
	(Galea et al. 2010)	cTBS	Left or right in separate groups	After learning	12-item explicit deterministic	Improved learning after 8 h consolidation
	(Tunovic et al. 2014) (Experiment 2)	cTBS	Right only	After learning	12-item explicit deterministic	Offline improvements after cTBS
	(Cohen and Robertson 2011) (Experiment 2)	1 Hz rTMS	Right only	After learning	12-item implicit deterministic	Decreased learning after 12 h consolidation by failing to prevent interference with a declarative task
	(Ambrus et al. 2020)	1 Hz rTMS	Bilaterally	Between learning blocks	8-item implicit probabilistic	Improved learning after 24 h consolidation
**Broca’s area**						
	(Clerget et al. 2012)	cTBS	Left only	Before learning	20-item implicit deterministic	Learning was prevented
**SMA**						
	(Pascual-Leone et al. 1996)	5 Hz rTMS	Not applicable	During learning	12-item implicit deterministic	No effect on learning
	(Wilkinson et al. 2010)	cTBS	Not applicable	Before learning	12-item implicit probabilistic	No effect on learning
	(Perez et al. 2008)	1 Hz rTMS	Not applicable	During learning	12-item implicit deterministic	Blocked intermanual transfer of the skill
**IPL**						
	(Robertson et al. 2001)	1 Hz rTMS	Contralateral to the dominant hand	Before learning	10-item implicit deterministic	No effect on learning
	(Rosenthal et al. 2009) (Experiment 1, 2)	cTBS	Right only	Before learning	12-item implicit probabilistic	Learning was disrupted in perceptual, but not in manual task
	(Breton and Robertson 2017)	1 Hz rTMS	Left only	After learning	12-item implicit, explicit deterministic	Blocked offline improvements in implicit, but not in explicit task
	(Clark et al. 2019)	cTBS	Left only	Before learning	12-item implicit, deterministic	No effect on learning
**Cerebellum**						
	(Torriero et al. 2004)	1 Hz rTMS	Left or right in separate groups	Before learning	12-item implicit, deterministic	Learning was disrupted

**Table 2:** Research design and power.

Design	Study	Type of Control Condition	N	N/Group
**Within-subjects**				
	(Pascual-Leone et al. 1996)	Absence of stimulation	7	
	(Robertson et al. 2001)	Active control (parietal cortex)	6	
	(Gann et al. 2021)	No control	19	
	(Steel et al. 2016)	Sham stimulation with placebo coil	22	
**Between-subjects**				
	(Robertson et al. 2005)	Sham stimulation with placebo coil, time, and site control	36	6
	(Breton and Robertson 2017)	Sham stimulation with placebo coil	67	12–16
	(Tunovic et al. 2014) (Experiment 2)	Intermediate TBS, localization control	24	12
	(Tunovic et al. 2014) (Experiment 3)	Intermediate TBS	24	12
	(Cohen and Robertson 2011) (Experiment 2)	Sham stimulation over vertex with placebo coil	40	10
	(Wilkinson et al. 2010)	Sham stimulation with 90° rotation of the coil	40	8
	(Rosenthal et al. 2009) (Experiment 1, 2)	Sham stimulation over vertex with placebo coil	24, 24	8, 8
	(Galea et al. 2010)	Active control (occipital cortex)	30	10
	(Ambrus et al. 2020)	Sham stimulation with 90° rotation of the coil	31	15 and 16
	(Clerget et al. 2012)	Active control (vertex)	17	8 and 9
	(Perez et al. 2008)	Sham stimulation with a second coil discharged in the air	33	11
	(Torriero et al. 2004)	Absence of stimulation	36	5–7
**Mixed design**				
	(Wilkinson et al. 2015)	Sham stimulation with placebo coil	40	20
	(Clark et al. 2019)	Sham stimulation with placebo coil	48	16

**Table 3:** Methodological details of the studies.

Study	Localization Technique	Intensity	Total Number of Pulses	Data Type of the Result
(Pascual-Leone et al. 1996)	Left or right DLPFC: scalp-based heuristics (5-cm rule; APB muscle) SMA: scalp-based heuristics (5-cm rule; TA muscle)	115% of MT	not available	RT
(Robertson et al. 2001)	Left or right DLPFC: scalp-based heuristics (5-cm rule; APB muscle) Left or right parietal cortex: scalp-based heuristics (P3 or P4 electrodes; 10–20 EEG system)	115% of MT	900	RT
(Robertson et al. 2005)	Left M1: APB muscle	90% of MT	600	Learning score based on RT
(Breton and Robertson 2017)	Left M1: APB muscle Left IPL: individual neuronavigation based on MRI coordinates, source of the exact coordinate is not traceable * (x = −48, y = −56, z = 46) * Note that the origin of the exact coordinate cannot be traced with high certainty (one of the two cited articles used different coordinates and the other did not involve neuroimaging)	90% of MT	600	Learning improvement based on RT
(Tunovic et al. 2014) (Experiment 2 and 3)	Right M1: FDI (reported as flexor dorsal interosseous muscle) Right DLPFC: individual neuronavigation based on MNI coordinates modified * from prior studies (x = 40, y = 32, z = 30) * Note that all of the cited prior studies in this reference reported coordinates at the left hemisphere (x = −40, y = 32, z = 30), and not at the right hemisphere	80% of AMT	600	Learning score based on RT
(Cohen and Robertson 2011) (Experiment 2)	M1: APB muscle Right DLPFC: individual neuronavigation based on MNI coordinates modified * from prior studies (x = 40, y = 32, z = 30) * Note that all of the cited prior studies in this reference reported coordinates at the left hemisphere (x = −40, y = 32, z = 30), and not at the right hemisphere	90% of MT	600	Learning score based on RT
(Rosenthal et al. 2009) (Experiment 1, 2)	Right AG: individual neuronavigation based on macroscopial anatomical identification of AG (posterior region of the IPL as the area directly adjacent to the dorsolateral projection of the superior temporal sulcus) Left or right M1: ‘hand’ muscle (no specific muscle is reported)	70% of RMT	300	Learning score based on RT
(Wilkinson et al. 2010)	Left M1: FDI muscle SMA: scalp-based heuristics (3 cm anterior and 0.5 cm to the left from Cz; 10–20 EEG system) Left DLPFC: scalp-based heuristics (5-cm rule; FDI muscle)	80% of AMT	600	Learning score based on RT
(Wilkinson et al. 2015)	Left M1: FDI muscle (spatial precision of inter-session targeting is controlled with neuronavigation)	80% of AMT	600	Learning score based on RT and accuracy
(Steel et al. 2016)	Left M1: FDI muscle (spatial precision of inter-session targeting is controlled with neuronavigation)	80% of AMT	600	Learning score based on RT
(Torriero et al. 2004)	Left or right lateral cerebellum: scalp-based heuristics (1 cm under and 3 cm left/right to the inion)	90% of MT	600	RT
(Perez et al. 2008)	SMA: scalp-based heuristics (3-cm rule; TA muscle)	80% of RMT	1200	RT
(Galea et al. 2010)	Left or right DLPFC: individual neuronavigation based on MNI coordinates modified from a previous publication (x = −40/+40, y = 28, z = 18)	80% of AMT	600	Learning improvement based on RT
(Clerget et al. 2012)	Left BA 44: individual neuronavigation based on MNI coordinates modified from prior studies (x = −43, y = 11, z = 16)	80% of RMT	600	RT
(Clark et al. 2019)	Left M1: FDI muscle Left parietal cortex: individual neuronavigation based on MNI coordinates from another prior study (x = −47, y = −68, z = 36)	70% of RMT	600	RT
(Gann et al. 2021)	Left DLPFC: individual neuronavigation based on subject-specific resting state functional connectivity analysis of the same participants	80% of AMT	600	RT and accuracy
(Ambrus et al. 2020)	Bilateral DLPFC: individual neuronavigation based of MNI coordinates from a previous meta-analysis (x = 37, y = 33, z = 32)	55% of MSO	3000 (1500/hemisphere)	Learning score based on RT

APB: abductor pollicis brevis, TA: tibialis anterior, FDI: first dorsal interosseous, AG: angular gyrus, IPL: inferior parietal lobule, MT: motor threshold, RMT: resting motor threshold, AMT: active motor threshold, MSO: maximum stimulator output, MEP: motor-evoked potential.

## Data Availability

No new data were created or analyzed in this study. Data sharing is not applicable to this article.

## References

[B1-jintelligence-11-00201] Aizenstein Howard J., Butters Meryl A., Clark Kristi A., Figurski Jennifer L., Stenger V. Andrew, Nebes Robert D., Reynolds Charles F., Carter Cameron S. (2006). Prefrontal and striatal activation in elderly subjects during concurrent implicit and explicit sequence learning. Neurobiology of Aging.

[B2-jintelligence-11-00201] Ambrus Géza Gergely, Vékony Teodóra, Janacsek Karolina, Trimborn Anna B. C., Kovács Gyula, Nemeth Dezso (2020). When less is more: Enhanced statistical learning of non-adjacent dependencies after disruption of bilateral DLPFC. Journal of Memory and Language.

[B3-jintelligence-11-00201] Andoh Jamila, Martinot Jean-Luc (2008). Interhemispheric compensation: A hypothesis of TMS-induced effects on language-related areas. European Psychiatry.

[B4-jintelligence-11-00201] Ashe James, Lungu Ovidiu V., Basford Alexandra T., Lu Xiaofeng (2006). Cortical control of motor sequences. Current Opinion in Neurobiology.

[B5-jintelligence-11-00201] Baetens Kris, Firouzi Mahyar, Van Overwalle Frank, Deroost Natacha (2020). Involvement of the cerebellum in the serial reaction time task (SRT) (Response to Janacsek et al.). NeuroImage.

[B6-jintelligence-11-00201] Bagnato Sergio, Curra Antonio, Modugno Nicola, Gilio Francesca, Quartarone Angelo, Rizzo Victor, Girlanda Paolo, Inghilleri Maurizio, Berardelli Alfredo (2005). One-hertz subthreshold rTMS increases the threshold for evoking inhibition in the human motor cortex. Experimental Brain Research.

[B8-jintelligence-11-00201] Beldarrain Marian Gomez, Gafman Jordan, de Velasco Ibone Ruiz, Pascual-Leone Alvaro, Garcia-Monco Juan (2002). Prefrontal lesions impair the implicit and explicit learning of sequences on visuomotor tasks. Experimental Brain Research.

[B7-jintelligence-11-00201] Beldarrain Marian Gómez, Grafman Jordan, Pascual-Leone Alvaro, Garcia-Monco Juan C. (1999). Procedural learning is impaired in patients with prefrontal lesions. Neurology.

[B9-jintelligence-11-00201] Bergmann Til Ole, Hartwigsen Gesa (2020). Inferring Causality from Noninvasive Brain Stimulation in Cognitive Neuroscience. Journal of Cognitive Neuroscience.

[B10-jintelligence-11-00201] Bergstrom Jennifer C. Romano, Howard James H., Howard Darlene V. (2012). Enhanced Implicit Sequence Learning in College-age Video Game Players and Musicians. Applied Cognitive Psychology.

[B11-jintelligence-11-00201] Berlot Eva, Popp Nicola J., Diedrichsen Jörn (2020). A critical re-evaluation of fMRI signatures of motor sequence learning. eLife.

[B12-jintelligence-11-00201] Beynel Lysianne, Powers John Paul, Appelbaum Lawrence Gregory (2020). Effects of repetitive transcranial magnetic stimulation on resting-state connectivity: A systematic review. NeuroImage.

[B13-jintelligence-11-00201] Boayue Nya Mehnwolo, Csifcsák Gábor, Aslaksen Per, Turi Zsolt, Antal Andrea, Groot Josephine, Hawkins Guy E., Forstmann Birte, Opitz Alexander, Thielscher Axel (2020). Increasing propensity to mind-wander by transcranial direct current stimulation? A registered report. European Journal of Neuroscience.

[B14-jintelligence-11-00201] Boyd Lara A., Linsdell Meghan A. (2009). Excitatory repetitive transcranial magnetic stimulation to left dorsal premotor cortex enhances motor consolidation of new skills. BMC Neuroscience.

[B15-jintelligence-11-00201] Breton Jocelyn, Robertson Edwin M. (2017). Dual enhancement mechanisms for overnight motor memory consolidation. Nature Human Behaviour.

[B16-jintelligence-11-00201] Buetefisch Cathrin M., Howard Cortney, Korb Christina, Haut Marc W., Shuster Linda, Pergami Paola, Smith Cheryl, Hobbs Gerald (2015). Conditions for enhancing the encoding of an elementary motor memory by rTMS. Clinical Neurophysiology.

[B17-jintelligence-11-00201] Bütefisch Cathrin M., Khurana Vikram, Kopylev Leonid, Cohen Leonardo G. (2004). Enhancing Encoding of a Motor Memory in the Primary Motor Cortex By Cortical Stimulation. Journal of Neurophysiology.

[B19-jintelligence-11-00201] Cao Na, Pi Yanling, Qiu Fanghui, Wang Yanqiu, Xia Xue, Liu Yu, Zhang Jian (2022). Plasticity changes in dorsolateral prefrontal cortex associated with procedural sequence learning are hemisphere-specific. NeuroImage.

[B18-jintelligence-11-00201] Cao Na, Pi Yanling, Liu Ke, Meng Haijiang, Wang Yanqiu, Zhang Jian, Wu Yin, Tan Xiaoying (2018). Inhibitory and facilitatory connections from dorsolateral prefrontal to primary motor cortex in healthy humans at rest—An rTMS study. Neuroscience Letters.

[B20-jintelligence-11-00201] Cardenas Valerie A., Bhat Jyoti V., Horwege Andrea M., Ehrlich Tobin J., Lavacot James, Mathalon Daniel H., Glover Gary H., Roach Brian J., Badran Bashar W., Forman Steven D. (2022). Anatomical and fMRI-network comparison of multiple DLPFC targeting strategies for repetitive transcranial magnetic stimulation treatment of depression. Brain Stimulation.

[B21-jintelligence-11-00201] Cárdenas-Morales Lizbeth, Nowak Dennis A., Kammer Thomas, Wolf Robert C., Schönfeldt-Lecuona Carlos (2010). Mechanisms and Applications of Theta-burst rTMS on the Human Motor Cortex. Brain Topography.

[B22-jintelligence-11-00201] Cash Robin F. H., Cocchi Luca, Lv Jinglei, Wu Yumeng, Fitzgerald Paul B., Zalesky Andrew (2021). Personalized connectivity-guided DLPFC-TMS for depression: Advancing computational feasibility, precision and reproducibility. Human Brain Mapping.

[B23-jintelligence-11-00201] Chouinard Philippe A., Leonard Gabriel, Paus Tomáš (2005). Role of the Primary Motor and Dorsal Premotor Cortices in the Anticipation of Forces during Object Lifting. Journal of Neuroscience.

[B24-jintelligence-11-00201] Clark Gillian M., Barham Michael P., Ware Anna T., Plumridge James M. A., O’Sullivan Bernadette, Lyons Kristie, Fitzgibbon Tegan, Buck Bree, Youssef George J., Ullman Michael T. (2019). Dissociable implicit sequence learning mechanisms revealed by continuous theta-burst stimulation. Behavioral Neuroscience.

[B25-jintelligence-11-00201] Clerget Emeline, Poncin William, Fadiga Luciano, Olivier Etienne (2012). Role of Broca’s Area in Implicit Motor Skill Learning: Evidence from Continuous Theta-burst Magnetic Stimulation. Journal of Cognitive Neuroscience.

[B27-jintelligence-11-00201] Cohen Daniel A., Pascual-Leone Alvaro, Press Daniel Z., Robertson Edwin M. (2005). Off-line learning of motor skill memory: A double dissociation of goal and movement. Proceedings of The National Academy of Sciences.

[B26-jintelligence-11-00201] Cohen Daniel A., Robertson Edwin M. (2011). Preventing interference between different memory tasks. Nature Neuroscience.

[B28-jintelligence-11-00201] Dahms Christiane, Brodoehl Stefan, Witte Otto W., Klingner Carsten M. (2020). The importance of different learning stages for motor sequence learning after stroke. Human Brain Mapping.

[B29-jintelligence-11-00201] Daselaar Sander M., Rombouts Serge A. R. B., Veltman Dick J., Raaijmakers Jeroen G. W., Jonker Cees (2003). Similar network activated by young and old adults during the acquisition of a motor sequence. Neurobiology of Aging.

[B30-jintelligence-11-00201] Daw Nathaniel D., Niv Yael, Dayan Peter (2005). Uncertainty-based competition between prefrontal and dorsolateral striatal systems for behavioral control. Nature Neuroscience.

[B31-jintelligence-11-00201] Dayan Eran, Censor Nitzan, Buch Ethan R., Sandrini Marco, Cohen Leonardo G. (2013). Noninvasive brain stimulation: From physiology to network dynamics and back. Nature Neuroscience.

[B32-jintelligence-11-00201] de Jesus Danilo R., de Souza Favalli Gabriela Pereira, Hoppenbrouwers Sylco S., Barr Mera S., Chen Robert, Fitzgerald Paul B., Daskalakis Zafiris J. (2014). Determining optimal rTMS parameters through changes in cortical inhibition. Clinical Neurophysiology.

[B33-jintelligence-11-00201] De Vries Meinou H., Barth Andre C. R., Maiworm Sandra, Knecht Stefan, Zwitserlood Pienie, Flöel Agnes (2010). Electrical Stimulation of Broca’s Area Enhances Implicit Learning of an Artificial Grammar. Journal of Cognitive Neuroscience.

[B34-jintelligence-11-00201] De Witte Sara, Klooster Debby, Dedoncker Josefien, Duprat Romain, Remue Jonathan, Baeken Chris (2018). Left prefrontal neuronavigated electrode localization in tDCS: 10–20 EEG system versus MRI-guided neuronavigation. Psychiatry Research: Neuroimaging.

[B35-jintelligence-11-00201] Dharani Krishnagopal (2015). The Biology of Thought: A Neuronal Mechanism in the Generation of Thought—A New Molecular Model.

[B36-jintelligence-11-00201] Dirnberger Georg, Novak Judith, Nasel Christian (2013). Perceptual Sequence Learning Is More Severely Impaired than Motor Sequence Learning in Patients with Chronic Cerebellar Stroke. Journal of Cognitive Neuroscience.

[B37-jintelligence-11-00201] Doppelmayr Michael, Pixa Nils Henrik, Steinberg Fabian (2016). Cerebellar, but not Motor or Parietal, High-Density Anodal Transcranial Direct Current Stimulation Facilitates Motor Adaptation. Journal of the International Neuropsychological Society.

[B38-jintelligence-11-00201] Doyon Julien, Gaudreau Danielle, Laforce Robert, Castonguay Martin, Bedard Paul J., Bedard Francois, Bouchard Jean-Pierre (1997). Role of the Striatum, Cerebellum, and Frontal Lobes in the Learning of a Visuomotor Sequence. Brain and Cognition.

[B39-jintelligence-11-00201] Duecker Felix, Sack Alexander T. (2015). Rethinking the role of sham TMS. Frontiers in Psychology.

[B40-jintelligence-11-00201] Ferrari Chiara, Cattaneo Zaira, Oldrati Viola, Casiraghi Letizia, Castelli Francesco, D’angelo Egidio, Vecchi Tomaso (2018). TMS Over the Cerebellum Interferes with Short-term Memory of Visual Sequences. Scientific Reports.

[B41-jintelligence-11-00201] Fitzgerald Paul B., Fountain Sarah, Daskalakis Zafiris J. (2006). A comprehensive review of the effects of rTMS on motor cortical excitability and inhibition. Clinical Neurophysiology.

[B42-jintelligence-11-00201] Foerde Karin, Poldrack Russ A. (2009). Procedural Learning in Humans. Encyclopedia of Neuroscience.

[B44-jintelligence-11-00201] Galea Joseph M., Vazquez Alejandro, Pasricha Neel, de Xivry Jean-Jacques Orban, Celnik Pablo (2011). Dissociating the Roles of the Cerebellum and Motor Cortex during Adaptive Learning: The Motor Cortex Retains What the Cerebellum Learns. Cerebral Cortex.

[B43-jintelligence-11-00201] Galea Joseph M., Albert Neil B., Ditye Thomas, Miall R. Chris (2010). Disruption of the Dorsolateral Prefrontal Cortex Facilitates the Consolidation of Procedural Skills. Journal of Cognitive Neuroscience.

[B45-jintelligence-11-00201] Gann Mareike A., King Bradley R., Dolfen Nina, Veldman Menno P., Chan Kimberly L., Puts Nicolaas A. J., Edden Richard A. E., Davare Marco, Swinnen Stephan P., Mantini Dante (2021). Hippocampal and striatal responses during motor learning are modulated by prefrontal cortex stimulation. NeuroImage.

[B46-jintelligence-11-00201] Gann Mareike A., Dolfen Nina, King Bradley R., Robertson Edwin M., Albouy Geneviève (2023). Prefrontal stimulation as a tool to disrupt hippocampal and striatal reactivations underlying fast motor memory consolidation. Brain Stimulation.

[B47-jintelligence-11-00201] Gentner Reinhard, Wankerl Katharina, Reinsberger Claus, Zeller Daniel, Classen Joseph (2008). Depression of Human Corticospinal Excitability Induced by Magnetic Theta-burst Stimulation: Evidence of Rapid Polarity-Reversing Metaplasticity. Cerebral Cortex.

[B48-jintelligence-11-00201] Glinski Benedikt (2021). Effects of Different Inhibitory Non-Invasive Brain Stimulation Protocols on Performance in a Motor Sequence Learning Task. Master’s Thesis.

[B49-jintelligence-11-00201] Goldsworthy Mitchell R., Hordacre Brenton, Rothwell John C., Ridding Michael C. (2021). Effects of rTMS on the brain: Is there value in variability?. Cortex.

[B50-jintelligence-11-00201] Gomez-Beldarrain Marian, Garcia-Monco Juan C., Rubio Berta, Pascual-Leone Alvaro (1998). Effect of focal cerebellar lesions on procedural learning in the serial reaction time task. Experimental Brain Research.

[B51-jintelligence-11-00201] Grafton Scott T., Hazeltine Eliot, Ivry Richard B. (1998). Abstract and Effector-Specific Representations of Motor Sequences Identified with PET. Journal of Neuroscience.

[B52-jintelligence-11-00201] Grafton Scott T., Hazeltine Eliot, Ivry Richard B. (2002). Motor sequence learning with the nondominant left hand. Experimental Brain Research.

[B53-jintelligence-11-00201] Hamada Masashi, Murase Nagako, Hasan Alkomiet, Balaratnam Michelle, Rothwell John C. (2013). The Role of Interneuron Networks in Driving Human Motor Cortical Plasticity. Cerebral Cortex.

[B54-jintelligence-11-00201] Hazeltine Eliot, Grafton Scott T., Ivry Richard (1997). Attention and stimulus characteristics determine the locus of motor- sequence encoding. A PET study. Brain.

[B55-jintelligence-11-00201] Hermsdorf Franz, Fricke Christopher, Stockert Anika, Classen Joseph, Rumpf Jost-Julian (2020). Motor Performance But Neither Motor Learning Nor Motor Consolidation Are Impaired in Chronic Cerebellar Stroke Patients. The Cerebellum.

[B57-jintelligence-11-00201] Hikosaka Okihide, Nakahara Hiroyuki, Rand Miya K., Sakai Katsuyuki, Lu Xiaofeng, Nakamura Kae, Miyachi Shigehiro, Doya Kenji (1999). Parallel neural networks for learning sequential procedures. Trends in Neurosciences.

[B56-jintelligence-11-00201] Hikosaka Okihide, Sakai Kuniyoshi, Miyauchi Satoru, Takino Ryousuke, Sasaki Yuka, Putz Benno (1996). Activation of human presupplementary motor area in learning of sequential procedures: A functional MRI study. Journal of Neurophysiology.

[B58-jintelligence-11-00201] Honda Manabu, Deiber Marie-Pierre, Ibánez Vicente, Pascual-Leone Alvaro, Zhuang Ping, Hallett Mark (1998). Dynamic cortical involvement in implicit and explicit motor sequence learning. A PET study. Brain.

[B59-jintelligence-11-00201] Hotermans Christophe, Peigneux Philippe, de Noordhout Alain Maertens, Moonen Gustave, Maquet Pierre (2008). Repetitive transcranial magnetic stimulation over the primary motor cortex disrupts early boost but not delayed gains in performance in motor sequence learning. European Journal of Neuroscience.

[B60-jintelligence-11-00201] Howard Darlene V., Howard James H., Japikse Karin, DiYanni Cara, Thompson Amanda, Somberg Rachel (2004). Implicit Sequence Learning: Effects of Level of Structure, Adult Age, and Extended Practice. Psychology and Aging.

[B61-jintelligence-11-00201] Huang Ying-Zu, Edwards Mark J., Rounis Elisabeth, Bhatia Kailash P., Rothwell John C. (2005). Theta Burst Stimulation of the Human Motor Cortex. Neuron.

[B62-jintelligence-11-00201] Huang Ying-Zu, Lu Ming-Kue, Antal Andrea, Classen Joseph, Nitsche Michael, Ziemann Ulf, Ridding Michael, Hamada Masashi, Ugawa Yoshikazu, Jaberzadeh Shapour (2017). Plasticity induced by non-invasive transcranial brain stimulation: A position paper. Clinical Neurophysiology.

[B63-jintelligence-11-00201] Janacsek Karolina, Nemeth Dezso (2013). Implicit sequence learning and working memory: Correlated or complicated?. Cortex.

[B64-jintelligence-11-00201] Janacsek Karolina, Nemeth Dezso (2015). The puzzle is complicated: When should working memory be related to implicit sequence learning, and when should it not? (Response to Martini et al.). Cortex.

[B65-jintelligence-11-00201] Janacsek Karolina, Nemeth Dezso (2022). Procedural Memory. The Cognitive Unconscious.

[B66-jintelligence-11-00201] Janacsek Karolina, Fiser József, Nemeth Dezso (2012). The best time to acquire new skills: Age-related differences in implicit sequence learning across the human lifespan. Developmental Science.

[B67-jintelligence-11-00201] Janacsek Karolina, Shattuck Kyle F., Tagarelli Kaitlyn M., Lum Jarrad A. G., Turkeltaub Peter E., Ullman Michael T. (2020). Sequence learning in the human brain: A functional neuroanatomical meta-analysis of serial reaction time studies. NeuroImage.

[B68-jintelligence-11-00201] Japikse Karin C., Negash Selam, Howard James H., Howard Darlene V. (2003). Intermanual transfer of procedural learning after extended practice of probabilistic sequences. Experimental Brain Research.

[B69-jintelligence-11-00201] Jayaram Gowri, Tang Byron, Pallegadda Rani, Vasudevan Erin V. L., Celnik Pablo, Bouffard Jason, Salomoni Sauro E., Mercier Catherine, Tucker Kylie, Roy Jean-Sébastien (2012). Modulating locomotor adaptation with cerebellar stimulation. Journal of Neurophysiology.

[B70-jintelligence-11-00201] Ji Gong-Jun, Yu Fengqiong, Liao Wei, Wang Kai (2017). Dynamic aftereffects in supplementary motor network following inhibitory transcranial magnetic stimulation protocols. NeuroImage.

[B71-jintelligence-11-00201] Juhasz Dora, Nemeth Dezso, Janacsek Karolina (2019). Is there more room to improve? The lifespan trajectory of procedural learning and its relationship to the between- and within-group differences in average response times. PLoS ONE.

[B72-jintelligence-11-00201] Jung Patrick, Ziemann Ulf (2009). Homeostatic and Nonhomeostatic Modulation of Learning in Human Motor Cortex. Journal of Neuroscience.

[B73-jintelligence-11-00201] Keele Steven W., Ivry Richard, Mayr Ulrich, Hazeltine Eliot, Heuer Herbert (2003). The cognitive and neural architecture of sequence representation. Psychological Review.

[B74-jintelligence-11-00201] Keramati Mehdi, Dezfouli Amir, Piray Payam (2011). Speed/Accuracy Trade-Off between the Habitual and the Goal-Directed Processes. PLoS Computational Biology.

[B75-jintelligence-11-00201] Khatibi Ali, Vahdat Shahabeddin, Lungu Ovidiu, Finsterbusch Jurgen, Büchel Christian, Cohen-Adad Julien, Marchand-Pauvert Veronique, Doyon Julien (2022). Brain-spinal cord interaction in long-term motor sequence learning in human: An fMRI study. NeuroImage.

[B76-jintelligence-11-00201] Kim Yun-Hee, Park Ji-Won, Ko Myoung-Hwan, Jang Sung Ho, Lee Peter K. W. (2004). Facilitative effect of high frequency subthreshold repetitive transcranial magnetic stimulation on complex sequential motor learning in humans. Neuroscience Letters.

[B77-jintelligence-11-00201] Klomjai Wanalee, Katz Rose, Lackmy-Vallée Alexandra (2015). Basic principles of transcranial magnetic stimulation (TMS) and repetitive TMS (rTMS). Annals of Physical and Rehabilitation Medicine.

[B78-jintelligence-11-00201] Kóbor Andrea, Janacsek Karolina, Hermann Petra, Zavecz Zzófia, Varga Virág, Csépe Valéria, Vidnyánszki Zoltán, Kovacs Gyula, Nemeth Dezso (2022). Finding pattern in the noise: Persistent implicit statistical knowledge impacts the processing of unpredictable stimuli. PsyArXiv.

[B79-jintelligence-11-00201] Lang Nicolas, Harms Jochen, Weyh Thomas, Lemon Roger N., Paulus Walter, Rothwell John C., Siebner Hartwig R. (2006). Stimulus intensity and coil characteristics influence the efficacy of rTMS to suppress cortical excitability. Clinical Neurophysiology.

[B80-jintelligence-11-00201] Lee Sang Wan, Shimojo Shinsuke, O’doherty John P. (2014). Neural Computations Underlying Arbitration between Model-Based and Model-free Learning. Neuron.

[B81-jintelligence-11-00201] Lieberman Matthew D. (2000). Intuition: A social cognitive neuroscience approach. Psychological Bulletin.

[B82-jintelligence-11-00201] Lum Jarrad A. G., Mills Andrea, Plumridge James M. A., Sloan Nicole P., Clark Gillian M., Hedenius Martina, Enticott Peter G. (2018). Transcranial direct current stimulation enhances retention of a second (but not first) order conditional visuo-motor sequence. Brain and Cognition.

[B83-jintelligence-11-00201] Lynch Charles J., Elbau Immanuel G., Ng Tommy H., Wolk Danielle, Zhu Shasha, Ayaz Aliza, Power Jonathan D., Zebley Benjamin, Gunning Faith M., Liston Conor (2022). Automated optimization of TMS coil placement for personalized functional network engagement. Neuron.

[B84-jintelligence-11-00201] Maeda Fumiko, Keenan Julian P., Tormos Jose M., Topka Helge, Pascual-Leone Alvaro (2000). Interindividual variability of the modulatory effects of repetitive transcranial magnetic stimulation on cortical excitability. Experimental Brain Research.

[B85-jintelligence-11-00201] McCalley Daniel M., Lench Daniel H., Doolittle Jade D., Imperatore Julia P., Hoffman Michaela, Hanlon Colleen A. (2021). Determining the optimal pulse number for theta burst induced change in cortical excitability. Scientific Reports.

[B86-jintelligence-11-00201] Meehan Sean K., Zabukovec Jeanie R., Dao Elizabeth, Cheung Katharine L., Linsdell Meghan A., Boyd Lara A. (2013). One hertz repetitive transcranial magnetic stimulation over dorsal premotor cortex enhances offline motor memory consolidation for sequence-specific implicit learning. European Journal of Neuroscience.

[B87-jintelligence-11-00201] Miller Earl K., Cohen Jonathan D. (2001). An Integrative Theory of Prefrontal Cortex Function. Annual Review of Neuroscience.

[B88-jintelligence-11-00201] Muellbacher Wolf, Ziemann Ulf, Wissel Joerg, Dang Nguyet, Kofler Markus, Facchini Stefano, Boroojerdi Babak, Poewe Werner, Hallett Mark (2002). Early consolidation in human primary motor cortex. Nature.

[B89-jintelligence-11-00201] Nemeth Dezso, Hallgató Emese, Janacsek Karolina, Sándor Timea, Londe Zsuzsa (2009). Perceptual and motor factors of implicit skill learning. NeuroReport.

[B91-jintelligence-11-00201] Nemeth Dezso, Janacsek Karolina, Polner Bertalan, Kovacs Zoltan Ambrus (2013). Boosting Human Learning by Hypnosis. Cerebral Cortex.

[B90-jintelligence-11-00201] Nemeth Dezso, Janacsek Karolina, Csifcsak Gabor, Szvoboda Gabor, Howard James H., Howard Darlene V. (2011). Interference between Sentence Processing and Probabilistic Implicit Sequence Learning. PLoS ONE.

[B92-jintelligence-11-00201] Nissen Mary Jo, Bullemer Peter (1987). Attentional requirements of learning: Evidence from performance measures. Cognitive Psychology.

[B93-jintelligence-11-00201] Ozdemir Recep A., Boucher Pierre, Fried Peter J., Momi Davide, Jannati Ali, Pascual-Leone Alvaro, Santarnecchi Emiliano, Shafi Mouhsin M. (2021). Reproducibility of cortical response modulation induced by intermittent and continuous theta-burst stimulation of the human motor cortex. Brain Stimulation.

[B94-jintelligence-11-00201] Page Matthew J., McKenzie Joanne E., Bossuyt Patrick M., Boutron Isabelle, Hoffmann Tammy C., Mulrow Cynthia D., Shamseer Larissa, Tetzlaff Jennifer M., Akl Elie A., Brennan Sue E. (2021). The PRISMA 2020 statement: An updated guideline for reporting systematic reviews. BMJ.

[B95-jintelligence-11-00201] Park Jungtak, Janacsek Karolina, Nemeth Dezso, Jeon Hyeon-Ae (2022). Reduced functional connectivity supports statistical learning of temporally distributed regularities. NeuroImage.

[B96-jintelligence-11-00201] Pascual-Leone Alvaro, Gates John R., Dhuna Anil (1991). Induction of speech arrest and counting errors with rapid-rate transcranial magnetic stimulation. Neurology.

[B97-jintelligence-11-00201] Pascual-Leone Alvaro, Wassermann Eric M., Grafman Jordan, Hallett Mark (1996). The role of the dorsolateral prefrontal cortex in implicit procedural learning. Experimental Brain Research.

[B98-jintelligence-11-00201] Pedraza Felipe, Farkas Bence C., Vékony Teodóra, Haesebaert Frederic, Phelipon Romane, Mihalecz Imola, Janacsek Karolina, Anders Royce, Tillmann Barbara, Plancher Gaën (2023). Evidence for a competitive relationship between executive functions and statistical learning. BioRxiv.

[B99-jintelligence-11-00201] Peigneux Philippe, Laureys Steven, Delbeuck Xavier, Maquet Pierre (2001). Sleeping brain, learning brain. The role of sleep for memory systems. NeuroReport.

[B100-jintelligence-11-00201] Pell Gaby S., Roth Yiftach, Zangen Abraham (2011). Modulation of cortical excitability induced by repetitive transcranial magnetic stimulation: Influence of timing and geometrical parameters and underlying mechanisms. Progress in Neurobiology.

[B101-jintelligence-11-00201] Perez Monica A., Tanaka Satoshi, Wise Steven P., Willingham Daniel T., Cohen Leonardo G. (2008). Time-Specific Contribution of the Supplementary Motor Area to Intermanual Transfer of Procedural Knowledge. Journal of Neuroscience.

[B102-jintelligence-11-00201] Polanía Rafael, Nitsche Michael A., Ruff Christian C. (2018). Studying and modifying brain function with non-invasive brain stimulation. Nature Neuroscience.

[B103-jintelligence-11-00201] Poldrack Russell A., Sabb Fred W., Foerde Karin, Tom Sabrina M., Asarnow Robert F., Bookheimer Susan Y., Knowlton Barbara J. (2005). The Neural Correlates of Motor Skill Automaticity. Journal of Neuroscience.

[B104-jintelligence-11-00201] Prashad Shikha, Du Yue, Clark Jane E. (2021). Sequence Structure Has a Differential Effect on Underlying Motor Learning Processes. Journal of Motor Learning and Development.

[B105-jintelligence-11-00201] Reber Arthur S. (1967). Implicit learning of artificial grammars. Journal of Verbal Learning and Verbal Behavior.

[B106-jintelligence-11-00201] Reber Arthur S. (1989). Implicit learning and tacit knowledge. Journal of Experimental Psychology: General.

[B107-jintelligence-11-00201] Robertson Edwin M. (2007). The Serial Reaction Time Task: Implicit Motor Skill Learning?. Journal of Neuroscience.

[B109-jintelligence-11-00201] Robertson Edwin M., Pascual-Leone Alvaro, Miall R. Chris (2004). Current concepts in procedural consolidation. Nature Reviews Neuroscience.

[B110-jintelligence-11-00201] Robertson Edwin M., Press Daniel Z., Pascual-Leone Alvaro (2005). Off-Line Learning and the Primary Motor Cortex. Journal of Neuroscience.

[B108-jintelligence-11-00201] Robertson Edwin M., Tormos Jose M., Maeda Fumiko, Pascual-Leone Alvaro (2001). The Role of the Dorsolateral Prefrontal Cortex during Sequence Learning is Specific for Spatial Information. Cerebral Cortex.

[B111-jintelligence-11-00201] Rosenthal Clive R., Roche-Kelly Emma E., Husain Masud, Kennard Christopher (2009). Response-Dependent Contributions of Human Primary Motor Cortex and Angular Gyrus to Manual and Perceptual Sequence Learning. Journal of Neuroscience.

[B112-jintelligence-11-00201] Rothkegel Holger, Sommer Martin, Paulus Walter (2010). Breaks during 5Hz rTMS are essential for facilitatory after effects. Clinical Neurophysiology.

[B113-jintelligence-11-00201] Ruitenberg Marit F. L., Verwey Willem B., Schutter Dennis J. L. G., Abrahamse Elger L. (2014). Cognitive and neural foundations of discrete sequence skill: A TMS study. Neuropsychologia.

[B114-jintelligence-11-00201] Sack Alexander T., Camprodon Joan A., Pascual-Leone Alvaro, Goebel Rainer (2005). The Dynamics of Interhemispheric Compensatory Processes in Mental Imagery. Science.

[B115-jintelligence-11-00201] Sack Alexander T., Kadosh Roi Cohen, Schuhmann Teresa, Moerel Michelle, Walsh Vincent, Goebel Rainer (2009). Optimizing Functional Accuracy of TMS in Cognitive Studies: A Comparison of Methods. Journal of Cognitive Neuroscience.

[B116-jintelligence-11-00201] Seidler Raphael D., Purushotham Arnie, Kim Seong-Gi, Ugurbil Kaamil, Willingham Daniel, Ashe James (2002). Cerebellum Activation Associated with Performance Change but Not Motor Learning. Science.

[B117-jintelligence-11-00201] Seidler Raphael D., Purushotham Arnie, Kim Seong-Gi, Ugurbil Kaamil, Willingham Daniel, Ashe James (2005). Neural correlates of encoding and expression in implicit sequence learning. Experimental Brain Research.

[B118-jintelligence-11-00201] Shanks David R. (2005). Implicit learning. Handbook of Cognition.

[B119-jintelligence-11-00201] Shimizu Takahiro, Hanajima Ritsuko, Shirota Yuichiro, Tsutsumi Ryosuke, Tanaka Nobuyuki, Terao Yasuo, Hamada Masashi, Ugawa Yoshikazu (2020). Plasticity induction in the pre-supplementary motor area (pre-SMA) and SMA-proper differentially affects visuomotor sequence learning. Brain Stimulation.

[B120-jintelligence-11-00201] Shin Jacqueline C., Ivry Richard B. (2003). Spatial and Temporal Sequence Learning in Patients with Parkinson’s Disease or Cerebellar Lesions. Journal of Cognitive Neuroscience.

[B121-jintelligence-11-00201] Simeoni Sara, Hannah Ricci, Sato Daisuke, Kawakami Michiyuki, Rothwell John, Gigli Gian Luigi (2016). Effects of Quadripulse Stimulation on Human Motor Cortex Excitability: A Replication Study. Brain Stimulation.

[B122-jintelligence-11-00201] Smalle Eleonore H. M., Möttönen Riikka (2023). Cognitive Development as a Piece of the Language Learning Puzzle. Cognitive Science.

[B123-jintelligence-11-00201] Smalle Eleonore H. M., Panouilleres Muriel, Szmalec Arnaud, Möttönen Riikka (2017). Language learning in the adult brain: Disrupting the dorsolateral prefrontal cortex facilitates word-form learning. Scientific Reports.

[B124-jintelligence-11-00201] Smalle Eleonore H. M., Daikoku Tatsuya, Szmalec Arnaud, Duyck Wouter, Möttönen Riikka (2022). Unlocking adults’ implicit statistical learning by cognitive depletion. Proceedings of the National Academy of Sciences of the United States of America.

[B125-jintelligence-11-00201] Smittenaar Peter, FitzGerald Thomas H. B., Romei Vincenzo, Wright Nicholas D., Dolan Raymond J. (2013). Disruption of Dorsolateral Prefrontal Cortex Decreases Model-Based in Favor of Model-free Control in Humans. Neuron.

[B126-jintelligence-11-00201] Song Sunbin, Howard James H., Howard Darlene V. (2007). Sleep Does Not Benefit Probabilistic Motor Sequence Learning. Journal of Neuroscience.

[B127-jintelligence-11-00201] Song Sunbin, Howard James H., Howard Darlene V. (2008). Perceptual sequence learning in a serial reaction time task. Experimental Brain Research.

[B128-jintelligence-11-00201] Steel Adam, Song Sunbin, Bageac Devin, Knutson Kristine M., Keisler Aysha, Saad Ziad S., Gotts Stephen J., Wassermann Eric M., Wilkinson Leonora (2016). Shifts in connectivity during procedural learning after motor cortex stimulation: A combined transcranial magnetic stimulation/functional magnetic resonance imaging study. Cortex.

[B129-jintelligence-11-00201] Thut Gregor, Pascual-Leone Alvaro (2010). A Review of Combined TMS-EEG Studies to Characterize Lasting Effects of Repetitive TMS and Assess Their Usefulness in Cognitive and Clinical Neuroscience. Brain Topography.

[B130-jintelligence-11-00201] Tiksnadi Amanda, Murakami Takenobu, Wiratman Winnugroho, Matsumoto Hideyuki, Ugawa Yoshikazu (2020). Direct comparison of efficacy of the motor cortical plasticity induction and the interindividual variability between TBS and QPS. Brain Stimulation.

[B131-jintelligence-11-00201] Torriero Sara, Oliveri Massimiliano, Koch Giacomo, Caltagirone Carlo, Petrosini Laura (2004). Interference of Left and Right Cerebellar rTMS with Procedural Learning. Journal of Cognitive Neuroscience.

[B132-jintelligence-11-00201] Tóth-Fáber Eszter, Nemeth Dezso, Janacsek Karolina (2023). Lifespan developmental invariance in memory consolidation: Evidence from procedural memory. PNAS Nexus.

[B133-jintelligence-11-00201] Tunovic Sanjin, Press Daniel Z., Robertson Edwin M. (2014). A Physiological Signal That Prevents Motor Skill Improvements during Consolidation. Journal of Neuroscience.

[B134-jintelligence-11-00201] Turi Zsolt, Lenz Maximilian, Paulus Walter, Mittner Matthias, Vlachos Andreas (2021). Selecting stimulation intensity in repetitive transcranial magnetic stimulation studies: A systematic review between 1991 and 2020. European Journal of Neuroscience.

[B135-jintelligence-11-00201] Turrigiano Gina (2012). Homeostatic Synaptic Plasticity: Local and Global Mechanisms for Stabilizing Neuronal Function. Cold Spring Harbor Perspectives in Biology.

[B136-jintelligence-11-00201] Uddén Julia, Ingvar Martin, Hagoort Peter, Petersson Karl Magnus (2017). Broca’s region: A causal role in implicit processing of grammars with crossed non-adjacent dependencies. Cognition.

[B137-jintelligence-11-00201] van der Graaf Ferdinand H. C. E., Maguire R. Paul, Leenders Klaus L., de Jong Bauke M. (2006). Cerebral activation related to implicit sequence learning in a Double Serial Reaction Time task. Brain Research.

[B138-jintelligence-11-00201] Veldman Menno P., Maurits Natasha M., Nijland M. A. M., Wolters Nicholas E., Mizelle John C., Hortobágyi Tibor (2018). Spectral and temporal electroencephalography measures reveal distinct neural networks for the acquisition, consolidation, and interlimb transfer of motor skills in healthy young adults. Clinical Neurophysiology.

[B140-jintelligence-11-00201] Verwey Willem B., Glinski Benedikt, Kuo Min-Fang, Salehinejad Mohammad Ali, Nitsche Michael A. (2022). Consolidation of motor sequence learning eliminates susceptibility of SMAproper to TMS: A combined rTMS and cTBS study. Experimental Brain Research.

[B139-jintelligence-11-00201] Verwey Willem B., Lammens Robin, Honk Jack van (2002). On the role of the SMA in the discrete sequence production task: A TMS study. Neuropsychologia.

[B141-jintelligence-11-00201] Walker Matthew P., Stickgold Robert (2005). It’s Practice, with Sleep, that Makes Perfect: Implications of Sleep-Dependent Learning and Plasticity for Skill Performance. Clinics in Sports Medicine.

[B143-jintelligence-11-00201] Wilkinson Leonora, Steel Adam, Mooshagian Eric, Zimmermann Trelawny, Keisler Aysha, Lewis Jeffrey D., Wassermann Eric M. (2015). Online feedback enhances early consolidation of motor sequence learning and reverses recall deficit from transcranial stimulation of motor cortex. Cortex.

[B142-jintelligence-11-00201] Wilkinson Leonora, Teo James T., Obeso Ignacio, Rothwell John C., Jahanshahi Marjan (2010). The Contribution of Primary Motor Cortex is Essential for Probabilistic Implicit Sequence Learning: Evidence from Theta Burst Magnetic Stimulation. Journal of Cognitive Neuroscience.

[B144-jintelligence-11-00201] Willingham Daniel B., Salidis Joanna, Gabrieli John D. E., Greeley Brian, Seidler Rachael D., Schambra Heidi M., Abe Mitsunari, Luckenbaugh David A., Reis Janine, Krakauer John W. (2002). Direct Comparison of Neural Systems Mediating Conscious and Unconscious Skill Learning. Journal of Neurophysiology.

[B145-jintelligence-11-00201] Yokoi Atsushi, Arbuckle Spencer A., Diedrichsen Jörn (2018). The Role of Human Primary Motor Cortex in the Production of Skilled Finger Sequences. Journal of Neuroscience.

[B146-jintelligence-11-00201] Yuan Peng, Raz Naftali (2014). Prefrontal cortex and executive functions in healthy adults: A meta-analysis of structural neuroimaging studies. Neuroscience & Biobehavioral Reviews.

[B147-jintelligence-11-00201] Zolnai Tamás, Dávid Dominika Réka, Pesthy Orsolya, Nemeth Marton, Kiss Mariann, Nagy Márton, Nemeth Dezso (2022). Measuring statistical learning by eye-tracking. Experimental Results.

